# Systems metabolic engineering for hydroxytyrosol production in *Escherichia coli*

**DOI:** 10.1128/aem.02455-25

**Published:** 2026-04-17

**Authors:** Wenjing Jiang, Zhichao Chen, Wenjing Fan, Changgeng Li, Lanxiao Li, Zichen Yu, Xu Li, Qingyang Xu

**Affiliations:** 1 College of Biotechnology, Tianjin University of Science & Technology 66345, Tianjin, People's Republic of China; 2 Key Laboratory of Industrial Fermentation Microbiology of the Ministry of Education, Tianjin University of Science & Technology 66345, Tianjin, People's Republic of China; Danmarks Tekniske Universitet, Kgs. Lyngby, Denmark

**Keywords:** hydroxytyrosol, *Escherichia coli*, metabolic engineering

## Abstract

Hydroxytyrosol (HT), the primary functional component of olive oil, presents significant challenges for microbial biosynthesis due to its inherent chemical properties and fermentation requirements. In this study, we describe the modular engineering of HT production in *Escherichia coli (E. coli*). We initially employed a highly efficient 4-hydroxyphenylacetic acid 3-monooxygenase (encoded by the *HpaBC* gene) from *E. coli*, phenylpyruvate decarboxylase (encoded by the *ARO10* gene), and alcohol dehydrogenase (encoded by the *ADH6* gene) from *Saccharomyces cerevisiae (S. cerevisiae*) to establish the *de novo* biosynthetic pathway for HT in *E. coli*. To enhance carbon flux toward HT production, we attenuated the competing metabolic pathway while reinforcing the synthetic route and optimized the gene copy numbers of key enzymes involved. Further strain engineering involved strengthening the membrane-bound pyridine nucleotide transhydrogenase (encoded by the *pntAB* gene) and incorporating a heterologous riboflavin biosynthesis pathway to improve the supply of the cofactors NADPH and FADH₂, culminating in the generation of the high-producing strain HT32-3. Then, a two-stage potential of hydrogen (pH) and two-stage dissolved oxygen (DO) control strategy was implemented to mitigate oxidative degradation of HT. In addition, a fed-batch supplementation process was developed to address the dependency of the key enzyme ARO10 on the cofactor vitamin B₁ (VB₁). These combined strategies enabled the achievement of a final HT titer of 9.22 g/L in a 5 L bioreactor. The present study provides a practical strategy for the modification of HT strains and a fermentation strategy, which lays the foundation for the development of engineered strains of high-value derivatives of tyrosine.

**IMPORTANCE:**

In this study, a strain with high hydroxytyrosol (HT) production capacity was constructed by means of metabolic engineering modification. A two-stage pH and two-stage DO fermentation strategy was developed based on its physicochemical properties. Combined with the VB_1_ replenishment strategy, the fermentation process of this strain was optimized, and the retention of HT was significantly improved, laying the foundation for the large-scale production of HT. This study explored the metabolic synthesis pathway and efficient fermentation strategy of HT, providing an innovative fermentation strategy and a practical strategy for the fermentation and production of HT and its related products.

## INTRODUCTION

Hydroxytyrosol (HT), also known as 3,4-dihydroxyphenylethanol, exhibits potent antioxidant properties. It participates in the regulation of multiple physiological functions in the human body (including anti-atherosclerosis [1], lipid regulation [2], and anti-osteoporosis [3]). It protects cellular DNA and enzymes from damage caused by reactive oxygen species (ROS), combining health and cancer prevention benefits (4). As the applications of HT continue to be explored, its market scale has steadily expanded and has been extended to the food and skin care sectors (5, 6). It is extracted primarily from olives. However, this extraction method relies heavily on seasonal raw materials, has low extraction rates, and requires lengthy production cycles. Industrial production commonly employs chemical synthesis and catalytic methods, which are expensive and cause significant pollution. In contrast, the microbial platform-based biosynthesis of HT is a promising commercial alternative (7).

Previous studies have attempted to synthesize HT in *Escherichia coli* (*E. coli)*. Chung et al. expressed the aromatic aldehyde synthase (AAS), tyrosine decarboxylase (TDC), tyramine oxidase (TYO), and 4-hydroxyphenylacetic acid 3-monooxygenase hydroxylase (HpaBC) to construct a strain that synthesized HT from tyrosine. The reported synthetic pathway is lengthy, yielding only 208 mg/L (8). Li et al. introduced the phenylpyruvate decarboxylase (ARO10), alcohol dehydrogenase (ADH6), and HpaBC to artificially construct a metabolic pathway extending from 4-hydroxyphenylpyruvate (4-HPP) to HT (9). This method significantly enhanced the efficiency of HT synthesis. Liu et al. screened for an efficient hydroxylase complex and achieved HT fermentation at 6.97 g/L (10). Wang et al. reported a titer of 9.87 g/L after introducing the L-amino acid deaminase (Laad) and enhancing cofactor recycling in the HpaBC (11). Xiong et al. performed fermentation using resorcinol, sodium pyruvate, and ammonium chloride as substrates. Coexpression of the tyrosine phenol lyase (TPL), Laad, α-ketoacid decarboxylase (KAD), aldehyde reductase (yahK), and glucose dehydrogenase (GDH) ultimately achieved a hydroxytoluene titer of 8.525 g/L (12).

**Fig 1:**
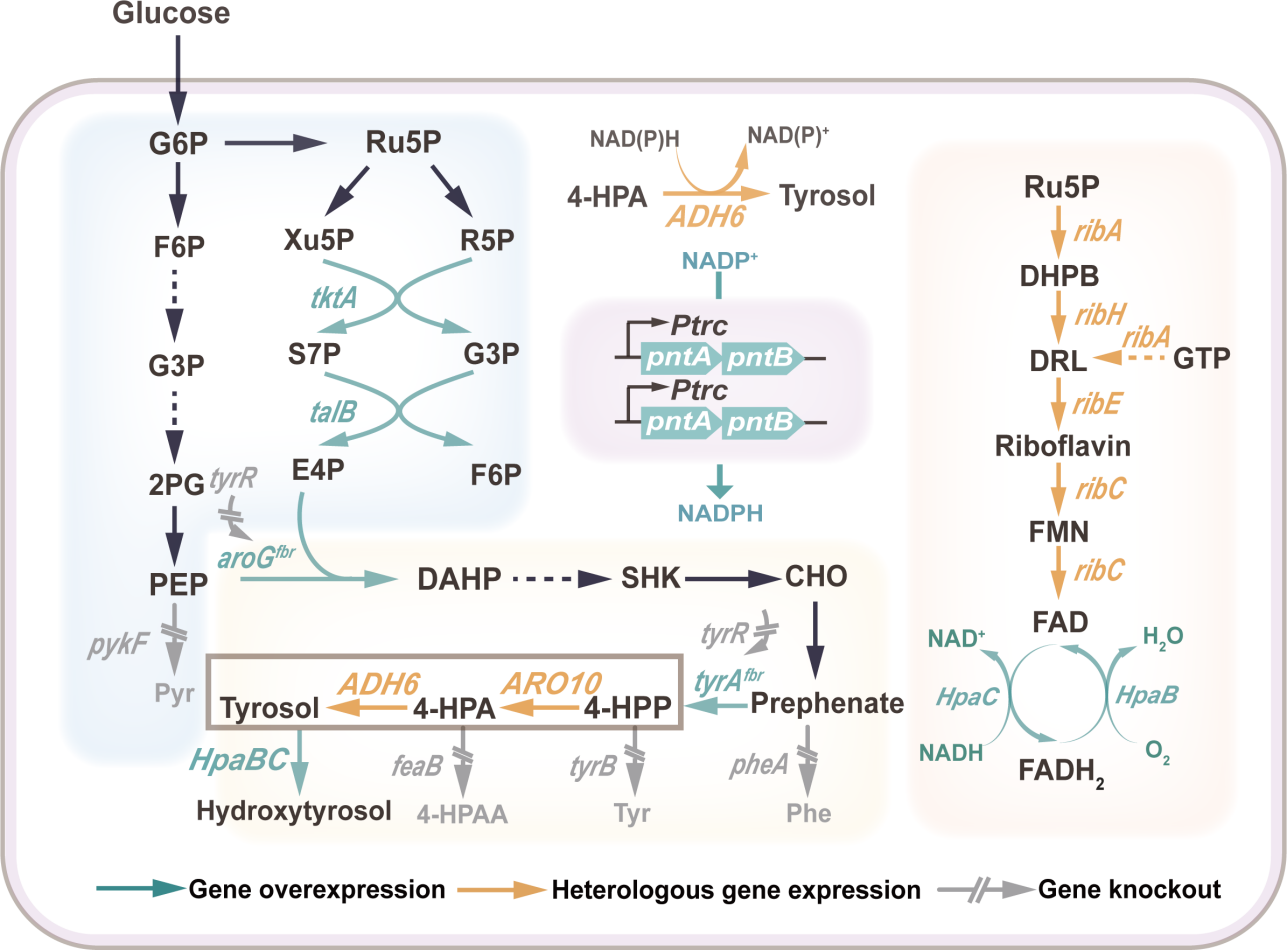
Metabolic pathway of HT biosynthesis and the metabolic engineering strategies employed in this study. The green arrow represents the overexpressed gene, the orange arrow represents the heterologous gene, and the gray arrow has two diagonal lines on it to represent the deleted gene. Abbreviations: G6P, glucose-6-phosphate; F6P, fructose-6-phosphate; G3P, glyceraldehyde 3-phosphate; 2PG, glycerate 2-phosphate; PEP, phosphoenolpyruvate; Pyr, pyruvate; Ru5P, ribulose-5-phosphate; Xu5P, xylulose-5-phosphate; R5P, ribose-5-phosphate; S7P, sedoheptulose 7-phosphate; G3P, glyceraldehyde 3-phosphate; E4P, erythrose 4-phosphate; DAHP, 3-deoxy-d-arabino-heptulosonate-7-phosphate synthase; SHK, shikimate; CHO, chorismate; 4-HPP, 4-hydroxyphenylpyruvate; Phe, phenylalanine; Tyr, L-tyrosine; 4-HPA, 4-hydroxyphenylacetaldehyde; 4-HPAA, 4-hydroxyphenylacetic acid; DHPB, L-3,4-dihydroxybutan-2-one 4-phosphate; DRL, 6,7-dimethyl-8-(d-ribityl)lumazine; GTP, guanosine 5'-triphosphate; FMN, riboflavin-5-phosphate; FAD, flavin adenine dinucleotide; *pykF*, pyruvate kinase; *tktA*, transketolase; *talB*, transaldolase; *tyrR*, DNA-binding transcriptional dual regulator; *aroG^fbr^*, 3-deoxy-7-phosphoheptulonate synthase; *tyrA^fbr^*, prephenate dehydrogenase; *pheA*, chorismate mutase/prephenate dehydratase; *tyrB*, tyrosine aminotransferase; *feaB*, phenylacetaldehyde dehydrogenase; *ARO10*, phenylpyruvate decarboxylase; *ADH6*, alcohol dehydrogenase; *HpaBC*, 4-hydroxyphenylacetic acid 3-monooxygenase; *ribA*, 3,4-dihydroxy 2-butanone 4-phosphate synthase; *ribH*, 6,7-dimethyl-8-ribityllumazine synthase; *ribE*, riboflavin synthase; *ribC*, riboflavin kinase/FMN adenylyltransferase.

Although HT has been widely studied, its complex metabolic pathway and high fermentation difficulty make industrial production still difficult to achieve. To address this limitation, we pursued systematic optimization of the HT biosynthetic pathway in *E. coli* (13). As outlined in Fig. 1, our engineering strategy encompassed the following: (i) introducing the HpaBC hydroxylation module, (ii) constructing the tyrosol synthesis pathway, (iii) enhancing the supply of the precursor 4-HPP, (iv) knocking down competing metabolic pathways, and (v) screening for the optimal gene copy numbers of *ARO10* and *ADH6*. Furthermore, to secure cofactor supply for the key enzymes ADH6 and HpaB, we augmented NADPH availability by overexpressing the *pntAB* gene and supplied the required FADH₂ for HpaB via a heterologous riboflavin biosynthesis pathway. Combined with HT’s own characteristics, this study targeted the development of a fermentation strategy combining two-stage pH and two-stage dissolved oxygen (DO). It not only provides a strategy for the *de novo* synthesis of HT in *E. coli* but also provides ideas for the synthesis and metabolism of other tyrosine derivatives.

## RESULTS

### Construction of the hydroxylation module

**Fig 2:**
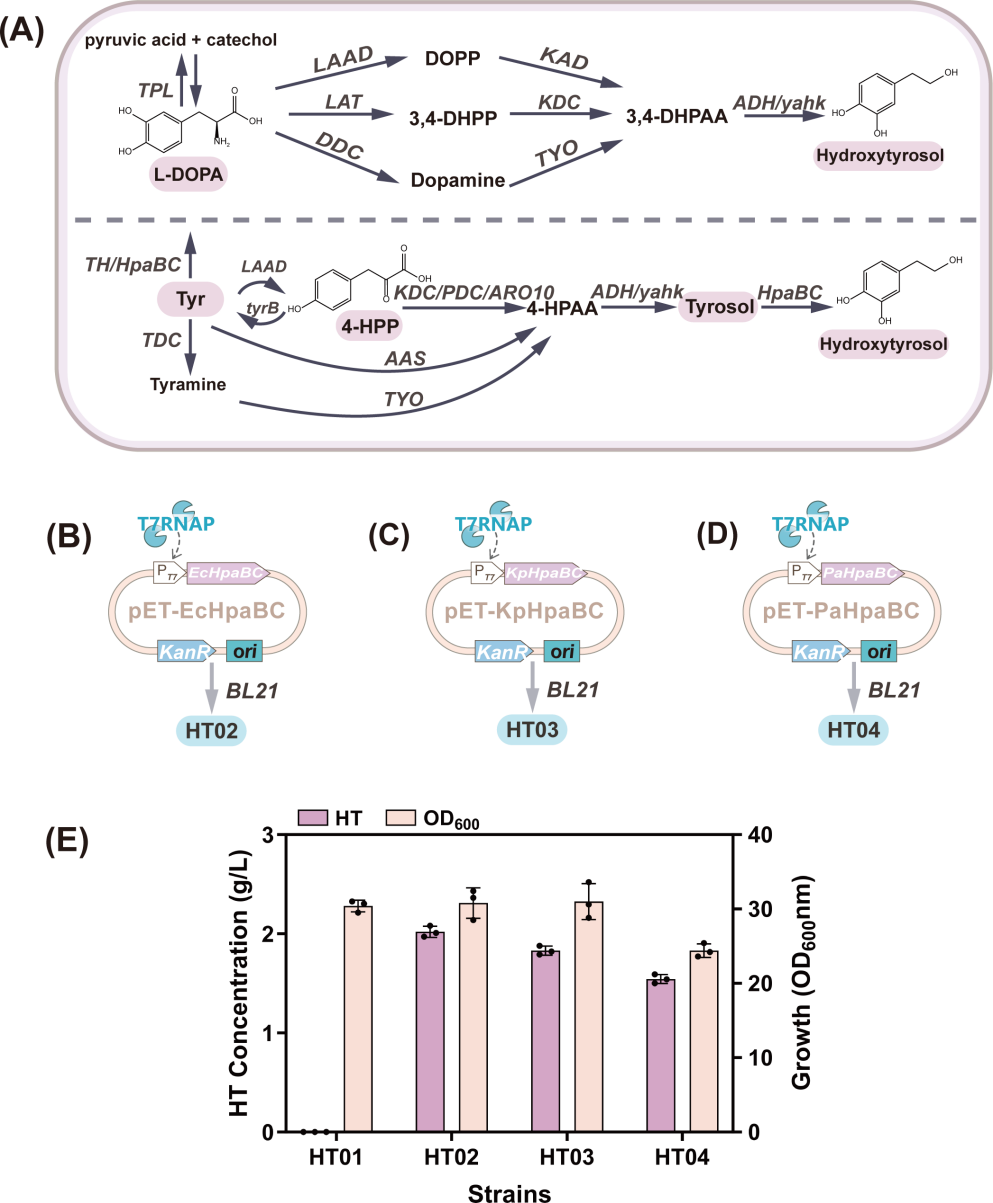
(**A**) Schematic diagram of the HT synthesis pathway. (**B**) Schematic diagram of *EcHpaBC* constructed in the pET-28a plasmid. (**C**) Schematic diagram of *KpHpaBC* constructed in the pET-28a plasmid. (**D**) Schematic diagram of *PaHpaBC* constructed in the pET-28a plasmid. (**E**) Effects of *HpaBC* from different sources on the strain’s production of HT and its biomass. Abbreviations: L-DOPA, 3,4-dihydroxyphenylalanine; DOPP, di-n-octyl phenylphosphonate; 3,4-DHPP, 3,4-dihydroxyphenylpyruvic acid; 3,4-DHPAA, 3,4-dihydroxyphenylacetaldehyde; Tyr, L-tyrosine; 4-HPP, 4-hydroxyphenylpyruvate; 4-HPAA, 4-hydroxyphenylacetic acid.

In the biological realm, two primary natural pathways exist for HT synthesis (Fig. 2A). Both pathways react sequentially with 3,4-dihydroxyphenylalanine (L-DOPA) and tyrosol as precursors to produce HT, both of which are tyrosine derivatives.

Recent studies have validated the tyrosol pathway as the most suitable route for HT biosynthesis in *E. coli* (14). This pathway generates HT from 4-HPP through the actions of decarboxylases, dehydrogenases, and hydroxylases. Among these, the catalytic activity of HpaBC in *E. coli* is a decisive factor in the heterologous synthesis of HT. Researchers have made significant efforts to screen and develop high-activity hydroxylases. In the present study, EcHpaBC from *E. coli* BL21, KpHpaBC from *Klebsiella pneumoniae*, and PaHpaBC from *Pseudomonas aeruginosa* were used to investigate the heterologous synthesis of HT in *E. coli* (15). The pET-28a vector and plasmids pET-EcHpaBC, pET-KpHpaBC, and pET-PaHpaBC (Fig. 2B, C and D) carrying *EcHpaBC*, *KpHpaBC*, and *PaHpaBC*, respectively, were introduced into *E. coli* BL21 to obtain strains HT01, HT02, HT03, and HT04. During shake-flask fermentation, isopropyl β-D-thiogalactopyranoside (IPTG) was added as an inducer when the OD_600_ of HT01, HT02, HT03, and HT04 reached 20. After 12 h of incubation, exogenous tyrosol was added as the substrate. Considering the stress effects of high tyrosol concentrations on *E. coli* (14), 4 g/L tyrosol was selected as the optimal substrate concentration. As shown in Fig. 2E, strains HT02, HT03, and HT04 catalyzed the conversion of tyrosol into HT. Notably, HT02 produced HT at a higher titer of 2.02 g/L, indicating that the EcHpaBC enzyme derived from *E. coli* was more conducive to tyrosol hydroxylation while maintaining robust cell growth. However, because tyrosol is primarily derived from natural olive extracts, its high cost makes exogenous addition impractical for industrial HT production. Consequently, the focus shifted to the *de novo* synthesis of HT in *E. coli*.

### Construction of the tyrosol synthesis pathway

**Fig 3:**
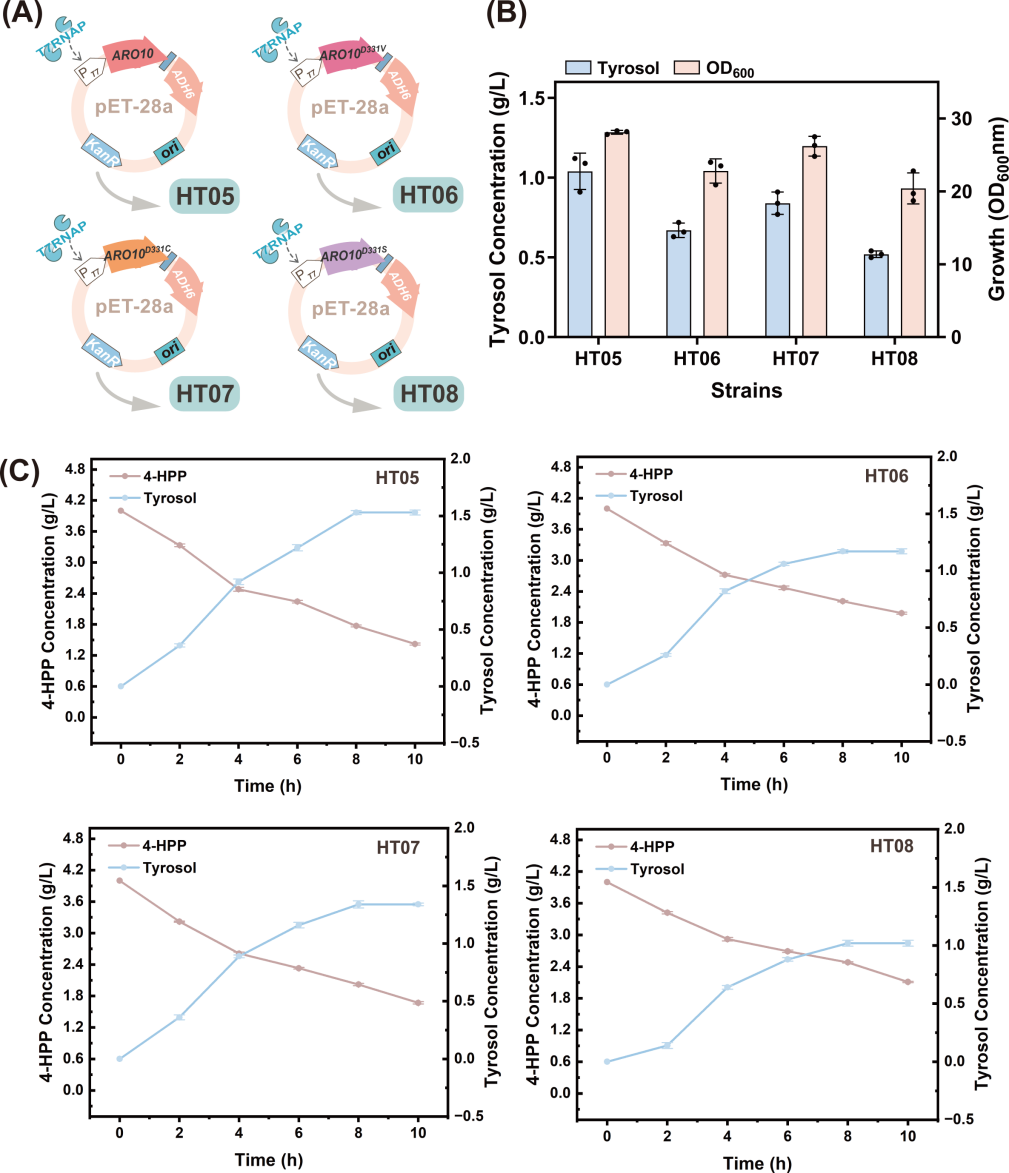
(**A**) Schematic diagram of *ARO10* and its mutants tandemly cloned with *ADH6* in the pET-28a plasmid. (**B**) Effects of different *ARO10* mutants on the strain’s production of HT and its biomass. (**C**) Titration changes in precursor 4-HPP and tyrosol.

In the tyrosol biosynthetic pathway engineered in *E. coli*, the conversion of 4-HPP to tyrosol represents the shortest and most efficient route (16). The phenylpyruvate decarboxylase gene *ARO10* and alcohol dehydrogenase gene *ADH6* from *S. cerevisiae* are frequently employed in engineering strategies for efficient tyrosol synthesis (9). When the aspartic acid residue at position 331 of the *ARO10* gene is mutated to serine, valine, or cysteine, the tyrosol titer significantly increases (14). Therefore, we constructed recombinant plasmids expressing different *ARO10* mutant genes and *ADH6* gene in tandem to evaluate the decarboxylation activity of ARO10 (Fig. 3A). Because the “xylose-induced T7 RNA polymerase-P*_T7_* promoter system” enables highly controllable expression of key enzyme genes (17, 18), the T7 RNA polymerase gene *T7RNAP* driven by P*xylF* was first integrated into the *lacI* site of *E. coli* W3110, forming the parental strain HT00. Subsequently, the recombinant plasmids were electrotransformed into HT00 cells to obtain the strains HT05, HT06, HT07, and HT08. After 8 h of shaking fermentation with 4 g/L 4-HPP as a precursor, the results (Fig. 3B) showed that during the continuous conversion of 4-HPP to tyrosol (Fig. 3C), strain HT05 produced the highest tyrosol yield (1.53 g/L) along with the highest OD_600_. In contrast, the mutants accumulated only 1.17 g/L, 1.34 g/L, and 1.02 g/L, indicating that the nonmutated *ARO10* gene conferred superior biosynthetic efficiency for tyrosol.

To convert tyrosol into HT, we introduced the previously screened hydroxylase gene *EcHpaBC* into plasmid pET-ARO10-ADH6 to yield plasmid pET-ARO10-ADH6-EcHpaBC. This plasmid was electrotransformed into strain HT00, resulting in strain HT09. Following the same 4-HPP addition, the HT titer reached 1.73 g/L. These results demonstrated that the co-introduction of the *ARO10*, *ADH6*, and *EcHpaBC* genes enabled HT synthesis in *E. coli*. However, HT accumulation remained low. To address this issue, rational modifications of the HT synthesis pathway are required (19).

### Enrichment of the 4-HPP precursor

**Fig 4:**
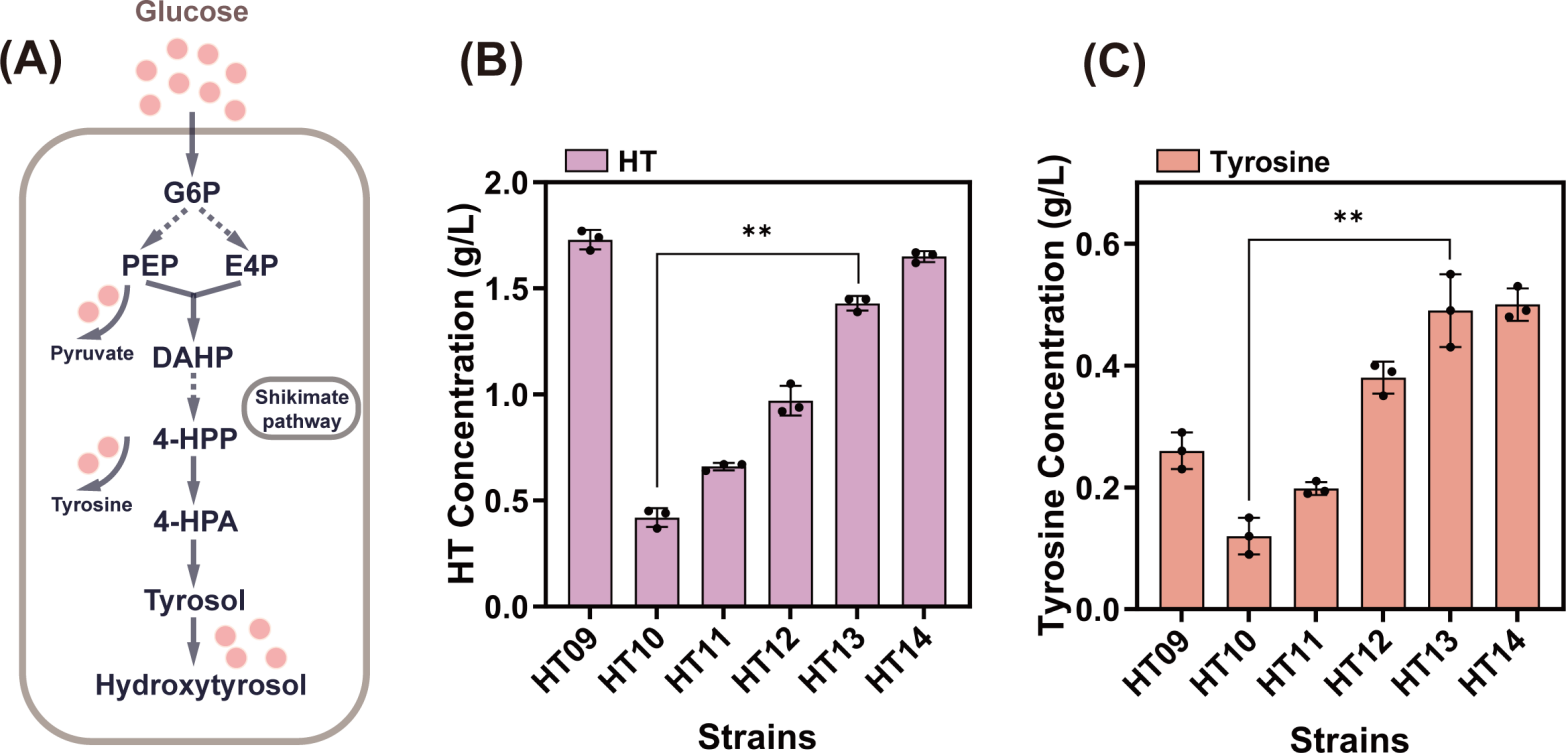
(**A**) Schematic diagram of carbon flux changes in the HT synthesis pathway of *E. coli*. (**B**) Effect of optimizing the 4-HPP synthesis pathway on the strain’s production of HT. (**C**) Effect of optimizing the 4-HPP synthesis pathway on the strain’s production of tyrosine. **, *P* < 0.01.

In *E. coli*, the carbon streams from phosphoenolpyruvate (PEP) in the glycolytic pathway and E4P in the pentose phosphate pathway converge and flow toward 4-HPP (Fig. 4A). Enhanced availability of PEP and E4P is considered crucial for increasing carbon flux in the shikimate pathway (20).

PEP is converted to pyruvate under the catalysis of pyruvate kinases (encoded by the *pykA* and *pykF* genes) (21) (Fig. 1). The *pykA* gene contributes minimally to pyruvate kinase activity (22). To reduce PEP loss, disruption of the *pykF* gene is typically used to decrease carbon flux into the tricarboxylic acid (TCA) cycle. Deletion of the *pykF* gene from the HT09 strain genome yielded the strain HT10. After shaking for 22 h, the HT titer reached 0.42 g/L. Conversely, overexpression of key enzymes in the non-oxidative pentose phosphate pathway, transketolase (encoded by *tktA*) and transaldolase (encoded by *talB*) (Fig. 1), significantly increased E4P availability (23). Overexpression of the *tktA* and *talB* genes in the HT10 genome resulted in strain HT11, which increased HT titer to 0.66 g/L—a 57.1% improvement over HT10.

3-Deoxy-D-arabino-heptulosonate-7-phosphate (DAHP) synthase catalyzes the condensation of PEP and E4P to form DAHP, which then enters the shikimate pathway (24). To efficiently direct PEP and E4P into the shikimate pathway, the feedback resistance gene *aroG^D146N^* (hereafter referred to as *aroG^fbr^*), driven by the trc promoter, was integrated into the genome of strain HT11, yielding strain HT12, which accumulated 0.97 g/L HT. The final product of the shikimate pathway, shikimic acid, is converted to 4-HPP in two steps catalyzed by chorismate mutase/prephenate dehydrogenase (encoded by the *tyrA* gene) (25, 26). To relieve the feedback inhibition of this gene by tyrosine, the feedback resistance gene *tyrA^M53I/A354V^* (hereafter referred to as *tyrA^fbr^*) was integrated into the HT12 genome to generate strain HT13. This resulted in a 47.4% increase in HT titer (1.43 g/L) compared to HT12, demonstrating that relieving the feedback regulation of *tyrA* enhances carbon flux toward 4-HPP. Considering the negative effect of the transcription regulator *tyrR* on the expression of DAHP synthase and prephenate dehydrogenase (27), the *tyrR* gene was deleted from the HT13 genome to obtain strain HT14. After 22 h of fermentation, the HT titer in HT14 increased to 1.65 g/L, demonstrating that the *tyrR* knockout positively influenced HT synthesis.

These results indicate that the overexpression of enzymes at key metabolic nodes in the 4-HPP synthesis pathway at the genomic level effectively promoted HT synthesis (Fig. 4B). Notably, small amounts of white crystals were detected on the side walls of the flask. High-performance liquid chromatography (HPLC) analysis confirmed that the crystals were composed of tyrosine (Fig. 4C). It is speculated that after 4-HPP enrichment, particularly following the integration of the *tyrA^fbr^* gene, the precursor 4-HPP was converted into tyrosine by the action of various aminotransferases (encoded by the *tyrB*, *hisC*, and *aspC* genes) (28, 29). The substantial accumulation of tyrosine then redirected the carbon flux toward the competing pathway for HT synthesis, the tyrosine pathway. Therefore, the next step was to investigate the effects of tyrosine and other byproducts generated during fermentation by strain HT14.

### Deleting competing pathways further enhances carbon flux

**Fig 5:**
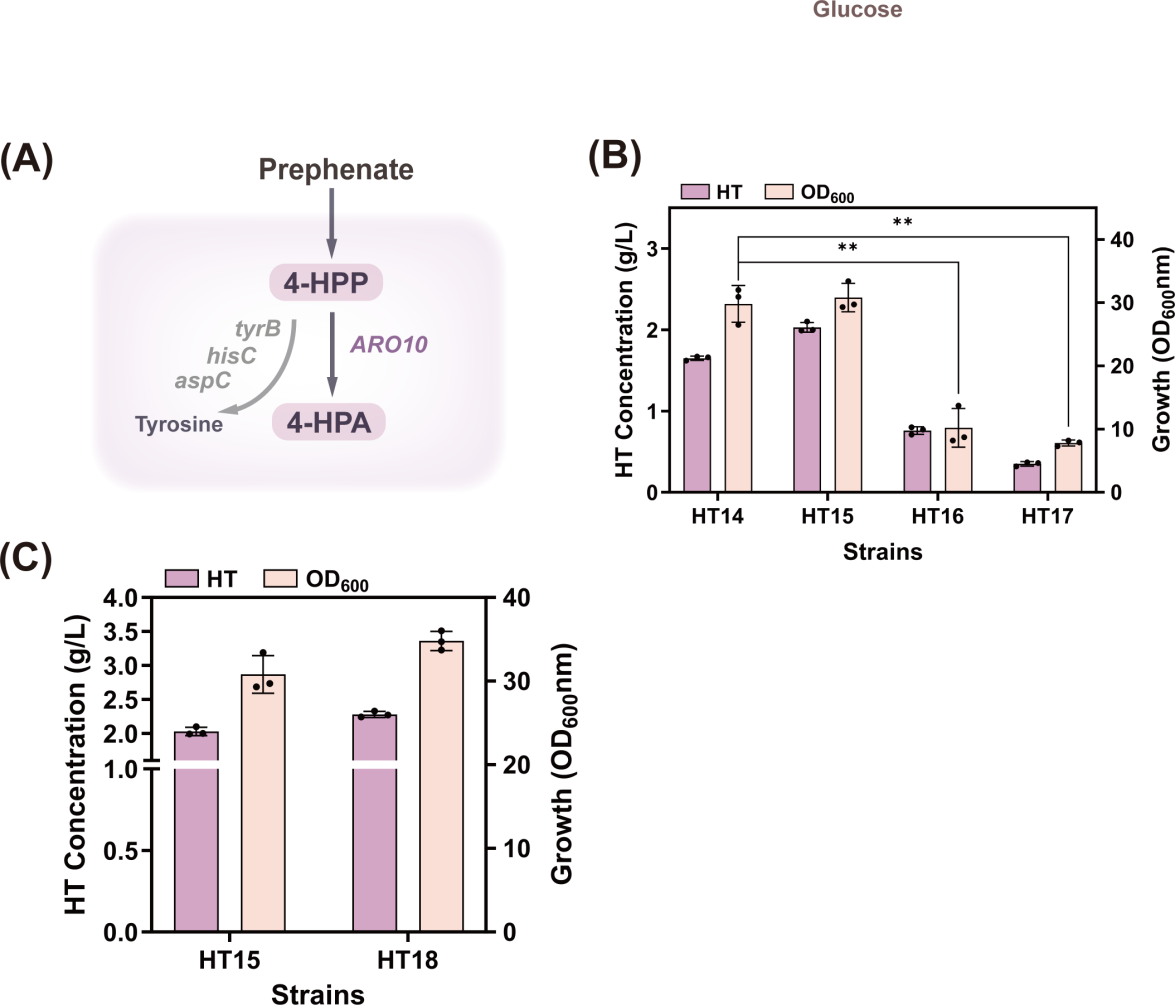
(**A**) The metabolic pathway for tyrosine production from 4-HPP. Abbreviations: *hisC*, histidinol-phosphate aminotransferase; *aspC*, aspartate aminotransferase. (**B**) Effects of *tyrB*, *hisC*, and *aspC* deletions on the strain’s production of HT and its biomass. (**C**) Effects of *pheA* and *feaB* deletions on the strain’s production of HT and its biomass. **, *P* < 0.01.

The direct deletion of genes in branch pathways is an effective strategy for blocking competing pathways and increasing product yields (30). In this study, *tyrB*, *hisC*, and *aspC* (Fig. 5A) were sequentially deleted from the HT14 genome to yield the strains HT15, HT16, and HT17, respectively. The results revealed (Fig. 5B) that the biomass of strains HT16 and HT17 was significantly reduced, decreasing by 66.9% and 74.7%, respectively, compared to that of strain HT15. This indicated that knocking out *hisC* and *aspC* severely impaired cell growth. Theoretically, *hisC* participates in histidine synthesis, whereas aspartic acid metabolism, mediated by *aspC*, plays a crucial role in coordinating chromosome replication, cell division, and cell growth (31). Therefore, knocking out these genes disrupts the amino acid metabolism within the cells, thereby affecting their growth. In contrast, the HT15 strain with only the *tyrB* gene knocked out exhibited normal growth, whereas the HT titer reached 2.03 g/L.

*pheA* encodes a prephenate dehydratase that redirects the carbon flux from prephenate to phenylpyruvate (32). *feaB* encodes a phenylacetaldehyde dehydrogenase that converts 4-HPA to 4-HPAA (33). Deletion of *pheA* and *feaB* from the HT15 genome yielded the strain HT18. After 22 h of shake-flask fermentation, strain HT18 showed undetectable levels of phenylalanine and 4-hydroxyphenylacetic acid, whereas HT accumulation reached 2.28 g/L (Fig. 5C) with robust cell growth. This demonstrated that controlling the carbon flux to the byproducts facilitated effective HT accumulation. However, it should be noted that residual tyrosine (0.74 g/L) remained in the shake flasks of strain HT18. Given the inability to eliminate tyrosine, we opted to enhance the expression of key enzymes in the heterologous HT synthesis pathway to “compete” for the precursor 4-HPP. Typically, the introduction of low-copy-number heterologous genes results in suboptimal expression within cells (34). Therefore, the copy number of heterologous genes was optimized to maximize the HT titer.

### Copy number screening of exogenous genes *ARO10* and *ADH6*

**Fig 6:**
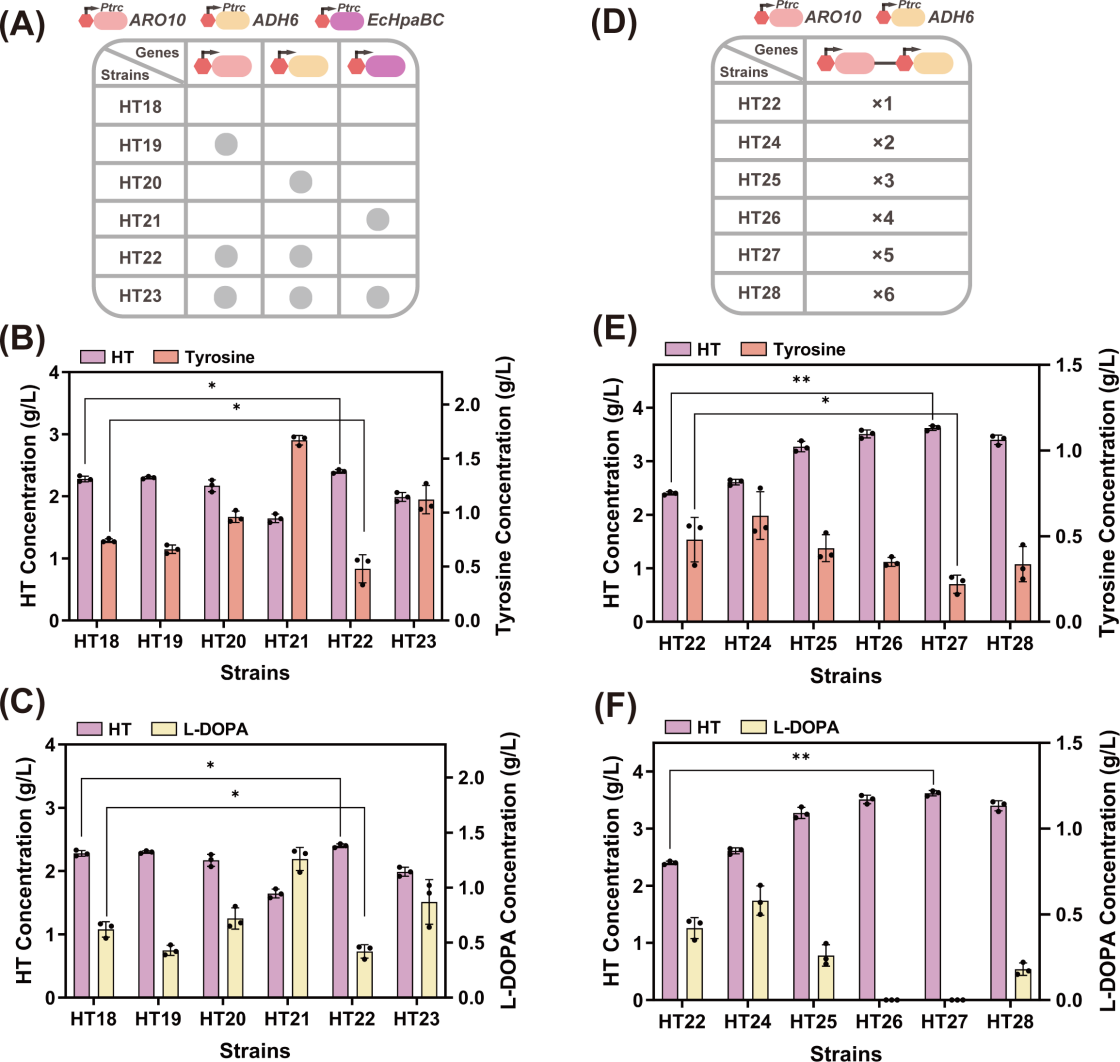
(**A**) Schematic representation of the integrated genes of strains HT18 to HT23. (**B**) Effects of introducing *ARO10*, *ADH6*, *EcHpaBC*, and their tandem genes *ARO10-ADH6* and *ARO10-ADH6-EcHpaBC* on the strain’s production of HT and tyrosine. (**C**) Effects of introducing *ARO10*, *ADH6*, *EcHpaBC*, and their tandem genes *ARO10-ADH6* and *ARO10-ADH6-EcHpaBC* on the strain’s production of HT and L-DOPA. (**D**) Schematic representation of the integration of different copy numbers of *ARO10-ADH6* on the strains. (**E**) Effects of *ARO10-ADH6* at different copy numbers on the strain’s production of HT and tyrosine. (**F**) Effects of *ARO10-ADH6* at different copy numbers on the strain’s production of HT and L-DOPA. *, 0.01 *< P <* 0.05; **, *P <* 0.01.

Owing to the instability of plasmid isolation (35), the introduction of plasmids alone cannot stably or effectively enhance the potency of HT. Genomic integration is a stable and persistent method for gene expression, and the combined expression levels achieved through genomic integration (36) and plasmid coexpression may exceed those achieved via single genomic integration or free plasmids alone (37). Therefore, we introduced heterologous genes into the genome while retaining the plasmid. *ARO10*, *ADH6*, *EcHpaBC*, *ARO10-ADH6*, and *ARO10-ADH6-EcHpaBC* tandem genes were integrated into the genome of HT18 to yield strains HT19, HT20, HT21, HT22, and HT23, respectively (Fig. 6A). Shake-flask fermentation results (Fig. 6B and C) showed that HT22 produced higher HT titers than the other four strains. Among these, strains HT21 and HT23, which had integrated the *EcHpaBC* gene, exhibited increased titers of tyrosine and L-DOPA byproducts. This indicated that integration of *EcHpaBC* into the genome negatively affected HT synthesis. By introducing double, triple, quadruple, quintuple, and sextuple copies of P*trc-ARO10-ADH6* into strain HT22, the strains HT24, HT25, HT26, HT27, and HT28 were generated (Fig. 6D). The results from the 22 h shake-flask cultures are shown in Fig. 6E and F. Compared with the control strain HT22, all five strains exhibited increased HT titers, with strain HT27 achieving the highest titer (3.62 g/L) and tyrosine residuals as low as 0.22 g/L. This demonstrated that the genomic multicopying of key genes significantly enhanced HT synthesis. Concurrently, HT26 exhibited HT titers comparable to those of HT27, whereas HT28’s titer (3.4 g/L) decreased by 6.1% relative to that of HT27. This phenomenon indicated that when copy numbers reached sufficiently high levels, further increases in HT potency became unattainable and may even negatively affect the synthesis. This may result from excessive heterologous gene expression in *E. coli,* depleting cellular amino acid pools, increasing the probability of protein misfolding, and imposing a substantial strain load (38). Thus, the optimal expression level for *ARO10* and *ADH6* in *E. coli* was five copies, and the HT27 strain was selected for subsequent studies.

### Construction of the ADH6 and HpaB cofactor recycling module

**Fig 7:**
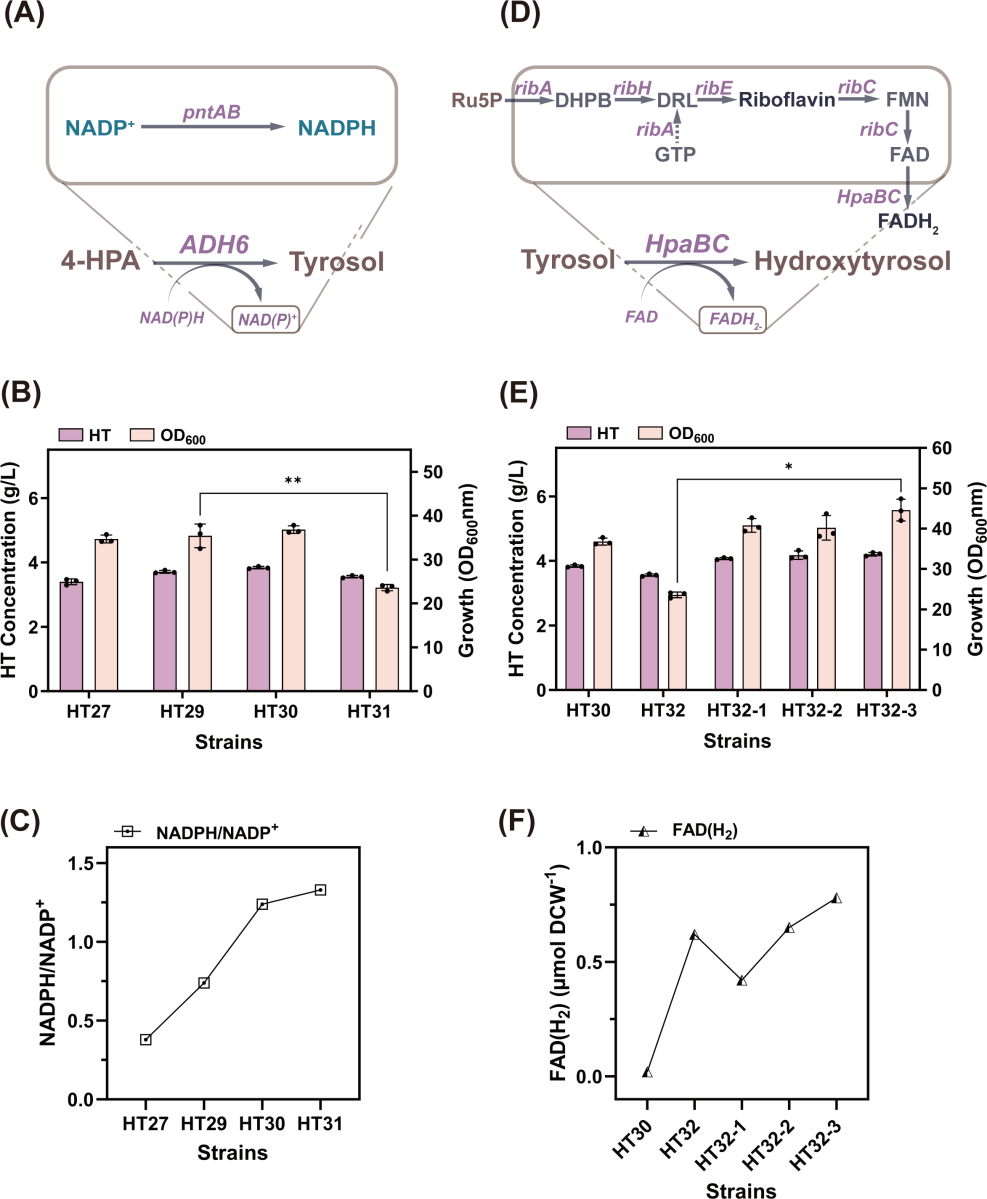
(**A**) The mechanism of action of *pntAB* in *E. coli*. (**B**) Effects of different *pntAB* copy numbers on strain performance and cell growth. (**C**) Intracellular NADPH/NADP^+^ levels in each strain. (**D**) The FADH₂ metabolic pathway in *Bacillus subtilis*. (**E**) Effects of three different promoters with varying strengths on strain performance and cell growth. (**F**) Intracellular FAD(H₂) levels in each strain. *, 0.01 < *P* < 0.05; **, *P* < 0.01.

As described in Section 3.2, the reaction catalyzed by the heterologously introduced ethanol dehydrogenase gene *ADH6* requires NADPH as a cofactor, wherein 1 mol of the aldehyde is reduced to alcohol using 1 mol of NADPH. The membrane-bound pyridine nucleotide transhydrogenase in *E. coli* (encoded by the *pntAB* gene) reduces NADP^+^ to NADPH by oxidizing NADH to NAD^+^(39) (Fig. 7A). To enhance NADPH utilization, we replaced the native pntAB promoter in HT27 with the trc promoter, yielding strain HT29. This modification increased the HT titer to 3.71 g/L. Subsequently, we increased the copy number of P*trc-pntAB* in HT29 to successively obtain strains HT30 and HT31. This resulted in HT yields of 3.84 g/L and 3.56 g/L, respectively, with HT31 exhibiting a significant decrease in OD_600_ (Fig. 7B). Intracellular reduced cofactor levels in the four strains revealed markedly higher NADPH/NADP^+^ ratios in HT30 and HT31 compared to HT27 and HT29 (Fig. 7C). These results indicated that optimizing NAD(P)H/NADP+ levels by enhancing pntAB expression positively affected HT synthesis. However, excessive NAD(P)H may disrupt the intrinsic cofactor balance in *E. coli*, leading to slow growth and adverse effects on HT production.

HpaB utilizes reduced FADH₂ to oxidize substrates, and its activity may be enhanced by increasing FADH₂ synthesis (11) (Fig. 7D). Endogenous FADH synthesis in *E. coli* depends on fFAD, the active coenzyme of riboflavin. To investigate this, we supplemented the existing medium with 0.2 g/L riboflavin to determine whether exogenous riboflavin positively affected HpaB enzyme activity. After 22 h of shake-flask fermentation, the HT titer increased to 3.92 g/L, representing a 2.1% improvement over HT30, demonstrating the efficacy of riboflavin. Furthermore, to reduce exogenous supplementation costs, this study engineered FADH₂ biosynthesis within *E. coli* to enhance FADH₂ availability. We heterologously introduced the riboflavin metabolic pathway from *Bacillus subtilis*, in which key intermediates such as FAD, FMN, and riboflavin required enhancement. Consequently, the four operons *ribA*, *ribH*, *ribE*, and *ribC* from *B. subtilis* were expressed in series and integrated into the HT30 genome. The resulting strain HT32 accumulated 4.12 g/L HT. Furthermore, promoter engineering was employed to select three distinct promoters for pathway enhancement, ranked from weakest to strongest: J23105 < J23101 < J23100. This yielded the strains HT32-1, HT32-2, and HT32-3, respectively. Shake-flask fermentation validation revealed that the strain incorporating the J23100 promoter produced the highest HT titer (4.21 g/L) as Fig. 7E. Cofactor quantification (Fig. 7F) showed significantly elevated intracellular FAD(H₂) levels in strain HT32-3 compared to the reference strain HT30. These results indicated that heterologous expression of the riboflavin pathway increased intracellular FADH₂ levels, with enhanced efficacy under strong promoter drive. This synthesis supplemented the cofactors required by HpaB, thereby strengthening the overall cofactor cycle of HpaBC and exerting a positive effect on its hydroxylation activity.

### Batch fermentation of hydroxytyrosol using feedstock additions

**Fig 8:**
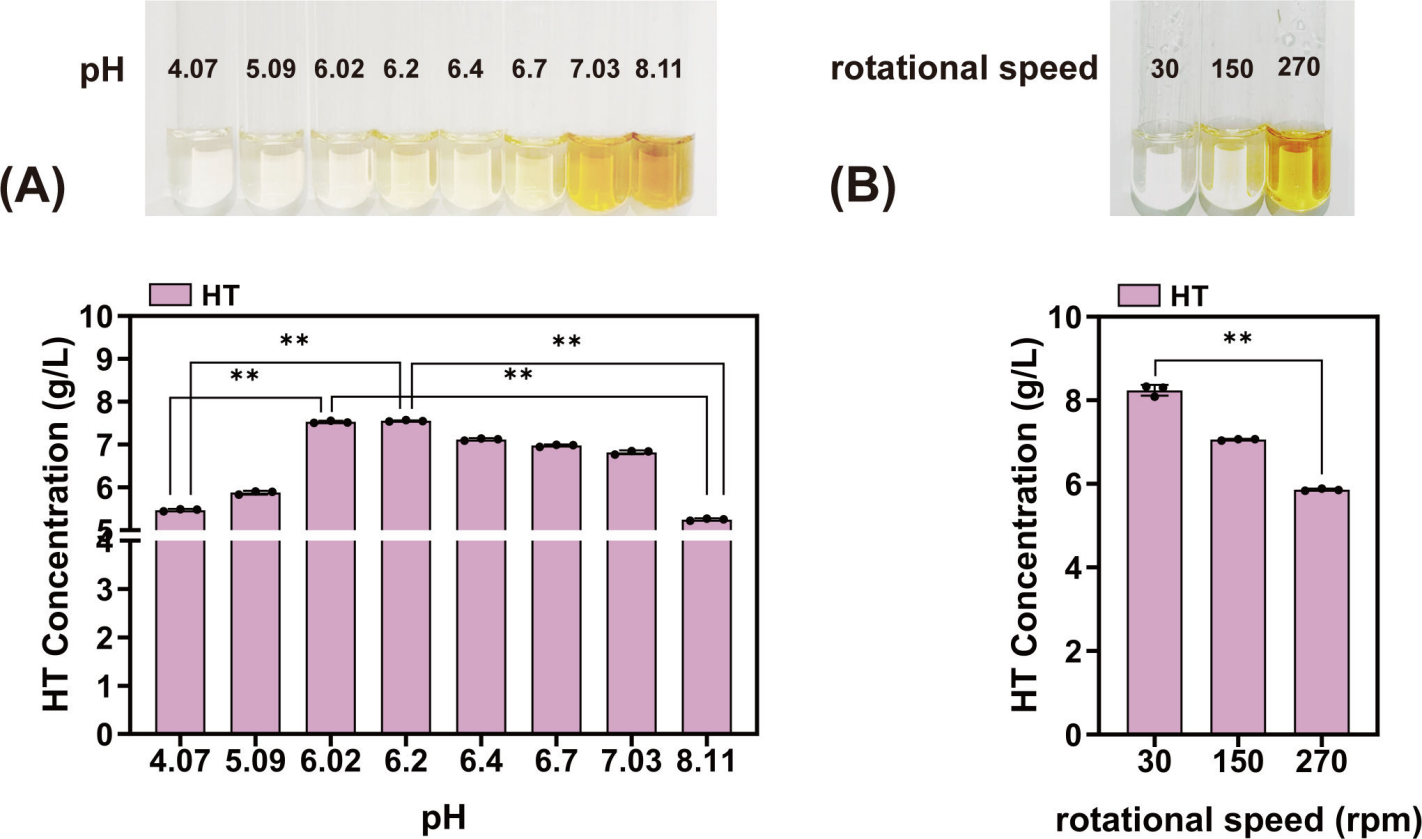
(**A**) Effect of pH on the color and stability of pure HT. (**B**) Effect of rotation speed on the stability of HT. **, *P* < 0.01.

The complexity of the fermentation process is another limiting factor for the efficient accumulation of HT. HT is a natural polyphenolic compound that contains two phenolic hydroxyl groups and one alcoholic hydroxyl group. Phenolic hydroxyl groups can act as hydrogen donors, thereby reducing the generation of free oxygen radicals. Considering these physicochemical properties, we hypothesized that the pH and dissolved oxygen (DO) during fermentation induce HT oxidation. Therefore, we employed a shaking flask to simulate fermentation conditions and first tested HT stability at different pH levels. Pure HT (10 g/L) was added to sterile deionized water, and the HT content was measured before and after fermentation under varying pH conditions. The results showed (Fig. 8A) that HT exhibited the lowest residual levels under weakly alkaline conditions. Visual inspection revealed that the pure HT solution darkened as the pH increased. This phenomenon confirmed that the pH factors induced the oxidation of HT.

Owing to the oxygen sensitivity of HT (40), high DO levels are unfavorable for its accumulation. To validate the effectiveness of low DO, we utilized 500 mL shaking flasks with a 30 mL liquid volume, adjusting the rotation speeds to 30, 150, and 270 rpm to simulate anaerobic, microaerophilic, and aerobic environments, respectively. Similarly, 10 g/L pure HT was added to sterile deionized water. Analysis of the HT content (Fig. 8B) revealed the highest residual levels at 30 rpm. This indicated that HT remained stable under anaerobic conditions, with concentrations 14.3% and 28.9% higher than those in the microaerophilic and aerobic environments, respectively. Visual inspection revealed that the pure compounds exhibited a darker color under the latter two conditions.

Fermentation of strain HT32-3 was conducted in a 5 L fermenter at 34℃ (pH 7.0, DO 30%–40%), yielding a titer of only 3.65 g/L in the scale-up test. This phenomenon indicated that HT accumulated poorly under neutral pH conditions with ample oxygen supply. To address this issue, this study proposes a two-stage pH and DO fermentation strategy that considers the combined effects of pH and DO.

### Two-stage pH regulation

**Fig 9:**
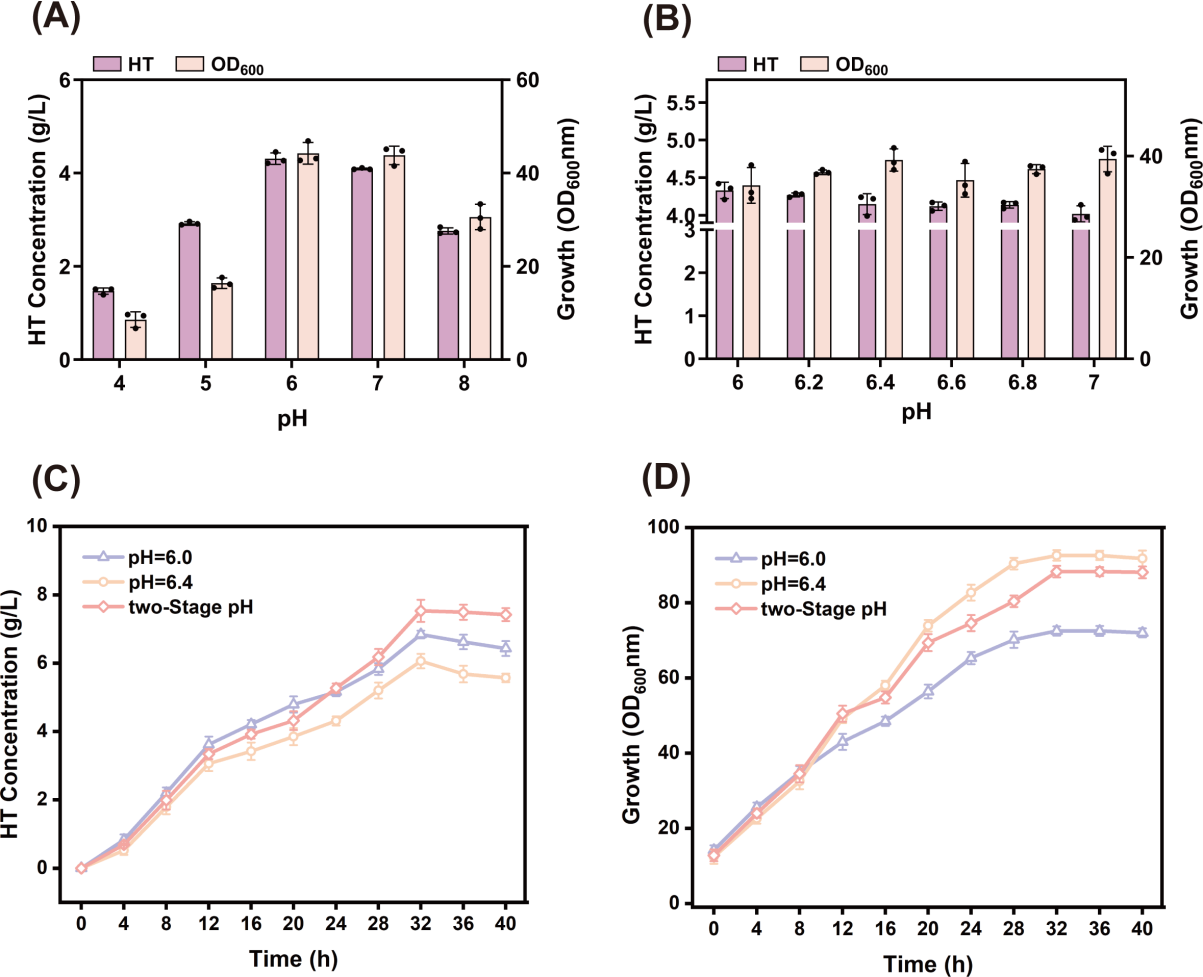
(**A**) Effect of five different pH levels on the strain’s production of HT and its biomass. (**B**) Effect of four different pH levels on the strain’s production of HT and its biomass. (**C**) Titration of HT during 40 h fermentation under different pH control conditions. (**D**) Biomass of HT during 40 h fermentation under different pH control conditions.

To screen for an optimal fermentation environment, five different pH values were used in preliminary experiments. The shaker fermentation results (Fig. 9A) showed higher HT titers at pH 6 and 7. Therefore, four different pH gradients were set between pH 6 and 7 to observe their effects on fermentation. The results (Fig. 9B) indicated that HT titers peaked at pH 6.0 (4.33 g/L), representing a 4.3% increase compared to pH 6.4 (4.15 g/L). At pH 6.0, the fermentation broth exhibited a gray color, which was distinctly different from the black broth observed at pH 7. This suggests that moderately lowering the pH is favorable for HT preservation. However, the OD_600_ was slightly lower at pH 6.0, which prevented optimal bacterial growth. Therefore, considering the advantages of fermentation at both pH levels, this study adopted a two-stage, pH-controlled fermentation process.

During the early fermentation stage, pH was maintained at 6.4 to promote optimal microbial growth. When the OD_600_ stabilized, the pH was lowered to 6.0 to facilitate HT preservation. Using strain HT32-3 in two-stage pH fermentation, the results showed that the logarithmic growth phase of cells persisted until 22 h into fermentation, after which it gradually entered a plateau phase. The OD_600_ reached its peak of 88.3 at 32 h, whereas the HT titer exhibited exponential growth after 4 h of fermentation. Control groups were fermented at a constant pH of 6.4 and 6.0 throughout (Fig. 9C and D). Fermentation at a constant pH of 6.4 showed vigorous strain growth but decreased the HT titer in the later stage. Fermentation at a constant pH of 6.0 enabled effective HT accumulation but resulted in slow strain growth. The two-stage pH-controlled fermentation process ensured normal microbial growth while promoting HT preservation. The maximum HT titer reached 7.53 g/L, representing a 24.3% and 10.1% increase compared to the fermentation indicators at pH 6.4 (6.06 g/L) and pH 6.0 (6.84 g/L), respectively.

### Oxygen “paradox” in fermentation

**Fig 10:**
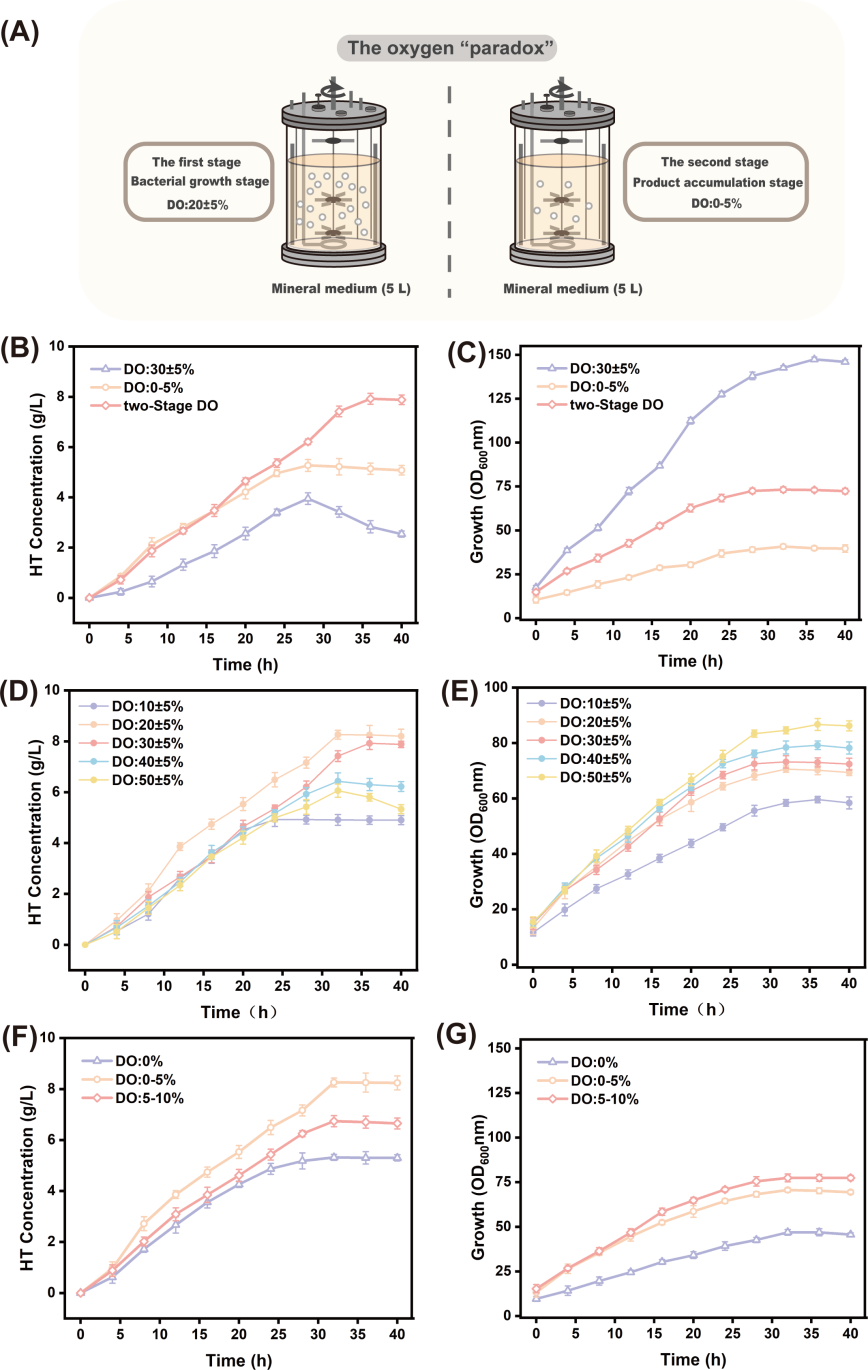
(**A**) Schematic diagram of the two-stage dissolved oxygen fermentation process. (**B**) Titration of HT during 40 h fermentation under different DO control conditions. (**C**) Biomass of HT during 40 h fermentation under different DO control conditions. (**D**) Titration of HT during 40 h fermentation under five DO conditions in the first stage. (**E**) Biomass of HT during 40 h fermentation under five DO conditions in the first stage. (**F**) HT titer during 40 h fermentation under five DO conditions in the second stage. (**G**) Biomass of HT during 40 h fermentation under five DO conditions in the second stage.

Maintaining the DO at a constant level throughout the process was detrimental to microbial growth (41), whereas a two-stage DO control strategy effectively balanced microbial growth with product accumulation (Fig. 10A). The optimal pH conditions were maintained. During the first fermentation stage (cell growth phase), DO was set at 30 ± 5%; in the second stage (product accumulation phase), aeration and agitation rates were reduced while maintaining DO at 0%–5%. Control groups included continuous aerobic fermentation (DO controlled at 30 ± 5%) and continuous oxygen-restricted fermentation (DO controlled at 0%–5%). The fermentation results (Fig. 10B) showed that the HT titer reached a maximum of 7.92 g/L in the two-stage fermentation method, representing increases of 100.5% and 50.3% compared to continuous full DO supply (3.95 g/L) and continuous DO restriction (5.27 g/L), respectively. Notably, during fermentation with restricted DO supply, the maximum OD_600_ value reached only 40.8 (Fig. 10C), representing a 79.4% and 261.3% decrease compared to two-stage fermentation (73.2) and fermentation with full oxygen supply (147.4), respectively. This demonstrated that a fermentation strategy with a restricted DO supply was unfavorable for HT production.

Further tests were conducted to evaluate HT accumulation under different DO gradients. The first-stage DO gradient was set to 10 ± 5%, 20 ± 5%, 30 ± 5%, 40 ± 5%, and 50 ± 5%, while the second-stage DO was maintained at 0%–5%. The highest titration (8.26 g/L) was achieved under a first-stage DO control of 20 ± 5% (Fig. 10D), with stable biomass (Fig. 10E). To maintain this condition, the second-stage DO was set to 0% (airflow rate: 0 VVM), 0%–5% (air flow rate: 0.175–0.375 VVM), and 5%–10% (air flow rate: 0.375–0.575 VVM). The results after 40 h of fermentation are shown in Fig. 10F and G. When the second-stage DO was set to 0%–5%, the titer and OD_600_ were significantly higher than those when DO was 0 or 5%–10%. Thus, the two-stage DO control process was advantageous for HT fermentation as it ensures both the oxygen required for the hydroxylation process and product stability.

### ThDP-dependent phenylpyruvate decarboxylase supplementation

**Fig 11:**
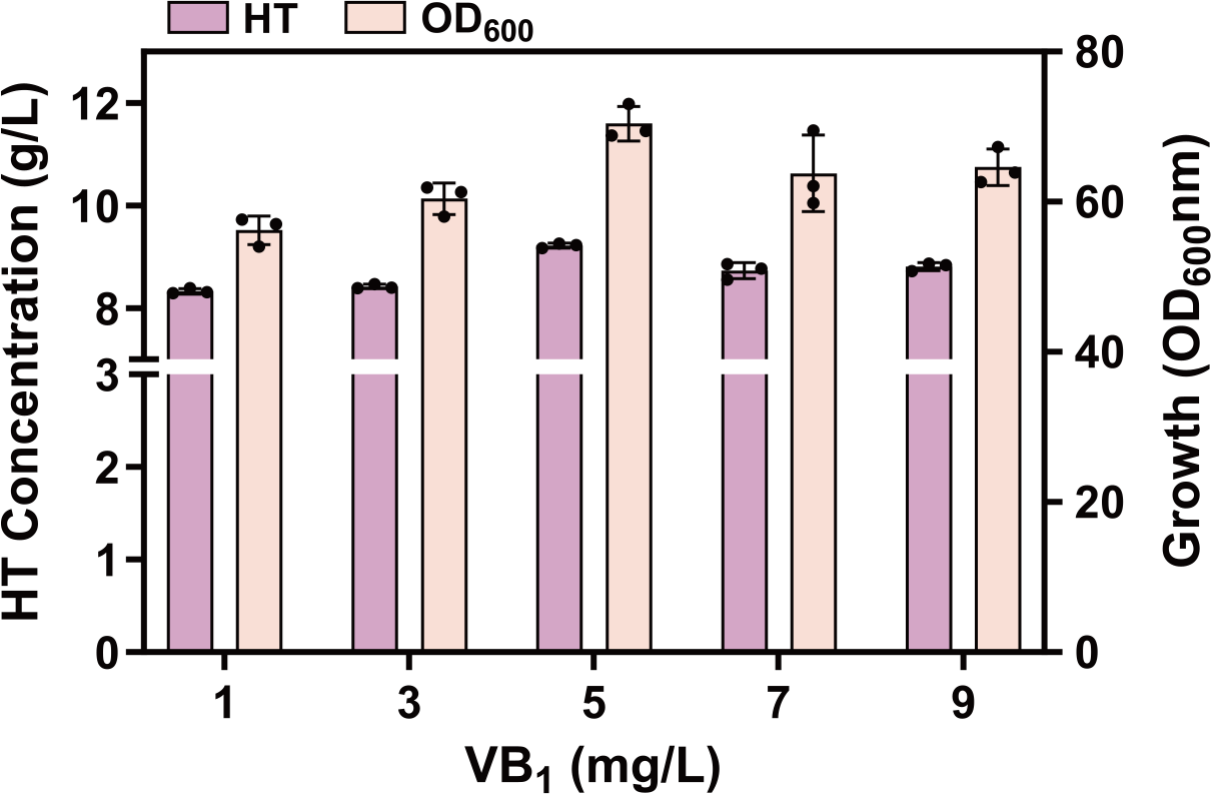
Effect of different VB_1_ addition rates on the strain’s production of HT and its biomass.

Genetic sequencing indicates that nearly all organisms possess both potential and actual ThDP-dependent enzymes (42). A total of 3 pyruvate decarboxylase isoforms, PDC1, PDC5, and PDC6, have been studied extensively. *S. cerevisiae* harbors a PDC-independent decarboxylase, a capability traceable to the ThDP-dependent *ARO10* gene (43). Thiamine diphosphate (ThDP), the biologically active form of vitamin B_1_, primarily mediates the formation and cleavage of carbon-carbon bonds near carbonyl groups (44). Considering the difficulty of *E. coli* accumulating VB_1_ endogenously, this study investigated the effects of exogenous VB_1_ supplementation on ARO10 expression after determining the optimal fermentation pH and DO conditions. Using HT32-3 as the experimental strain, VB_1_ was sequentially added as a cofactor at concentrations of 1, 3, 5, 7, and 9 mg/L. The fermentation results (Fig. 11) showed that the HT titer peaked at 9.22 g/L with a maximum OD_600_ at a VB_1_ concentration of 5 mg/L. At 7 mg/L and 9 mg/L VB_1_, the HT titer remained stable (8.73 g/L and 8.81 g/L, respectively), confirming the efficacy of exogenous VB_1_ supplementation. This study established an optimal dosage of 5 mg/L, marking the first demonstration that exogenous VB_1_ promoted HT accumulation.

## DISCUSSION

With the increasingly widespread application of HT in the food and biopharmaceutical fields, establishing an efficient microbial cell factory for HT production to meet the expanding market demand is essential. *E. coli* has emerged as the optimal choice for microbial fermentation because of its well-defined genetic background, established gene editing methods, and ease of cultivation. However, HT production in *E. coli* faces challenges such as low titers and poor heterologous gene expression. To overcome these limitations, we used modular engineering to optimize the HT synthesis pathway. First, highly efficient hydroxylase genes were identified. Subsequently, a metabolic pathway for tyrosol synthesis was heterologously introduced into *E. coli*, successfully establishing a *de novo* HT synthesis pathway. This pathway was further optimized using precursor 4-HPP enrichment, competitive pathway knockout, heterologous gene copy number screening, and cofactor supply optimization (45, 46). Finally, the genetically engineered strain HT32-3 was used for a 5 L pilot-scale fermentation study, where fermentation parameters, such as pH, DO, and VB_1_ dosage, were optimized. The optimized strain achieved a titer at the higher end of the currently reported levels, demonstrating its significant industrial application potential.

Tyrosol requires conversion to HT via hydroxylation by HpaBC (47). In the present study, HpaBC enzymes from *E. coli*, *K. pneumoniae*, and *P. aeruginosa* were screened (15). The *EcHpaBC* from *E. coli* exhibited the highest tyrosol conversion efficiency (50.5%). However, the addition of exogenous tyrosol imposes a significant burden on industrial production. Therefore, *ARO10* and *ADH6* were introduced heterologously to construct a tyrosol synthesis pathway. Screening of *ARO10* mutants revealed that strains harboring a nonmutated *ARO10* gene with codon optimization produced higher levels of HT. This phenomenon likely resulted from a single amino acid mutation that altered the protein structure, destabilizing the protein and impairing its normal expression (48). Furthermore, variations in medium conditions such as pH, temperature, ionic strength, and cofactor concentration can adversely affect the enzyme activity. Subsequently, the availability of the direct precursor 4-HPP was enhanced by optimizing key genes and eliminating competing pathways. This included the deletion of the *pykF* (49) gene and overexpression of the *tktA* and *talB* genes to increase PEP and E4P availability (50), raising the HT titer to 0.66 g/L. Subsequently, the *tyrR* gene was deleted (27), and *aroG^fbr^* and *tyrA^fbr^* were introduced to enhance metabolic flux in the shikimate pathway (51). This resulted in a 116.7% increase in the HT content. However, tyrosine accumulation was an unintended consequence. We investigated the amino acid transferases involved in tyrosine synthesis: *tyrB*, *hisC*, and *aspC*. Deletion of *hisC* and *aspC* caused a significant decrease in OD_600_. This disruption of amino acid metabolism severely inhibited growth (31). Therefore, we knocked out only the *tyrB* gene while simultaneously deleting the *pheA* (52) and *feaB* (53) genes in the branched metabolic pathway. This resulted in an increase in the HT titer to 2.28 g/L. However, tyrosine residues remained relatively high at this stage. Typically, enhancing the expression of key downstream enzymes may be a more effective strategy to redirect carbon flux toward HT production. Therefore, we integrated the key enzyme genes *ARO10*, *ADH6*, and *EcHpaBC* and their tandem genes, *ARO10-ADH6* and *ARO10-ADH6-EcHpaBC*, into the chromosome. Strain HT27 carrying the *ARO10-ADH6* tandem gene had the highest HT titer (3.62 g/L). Further increasing the copy number of *ARO10-ADH6* revealed that the five-copy P*trc-ARO10-ADH6* construct substantially elevated the HT titer (from 2.28 g/L to 3.62 g/L). Subsequent analysis revealed that six copies caused a decrease in the titer, likely resulting from excessive heterologous gene expression, leading to protein folding errors and imposing a growth burden on cells.

Because the reaction catalyzed by ADH6 requires NADPH as a cofactor, the pyrimidine nucleoside transhydrogenase encoded by *pntAB* can precisely drive the reduction of NADP^+^ to NADPH (39). To enhance NADPH synthesis, *pntAB* genes were overexpressed, increasing HT titers by 6.1% while raising the NADPH/NADP^+^ ratio from 0.38 to 1.24. Additionally, the activity of HpaB within the EchpaBC hydroxylase complex increased with FADH_2_ production, making FADH_2_ accumulation a significant factor. Through the heterologous introduction of the *ribA*, *ribH*, *ribE*, and *ribC* operons from *B. subtilis* and the selection of promoters with varying strengths for regulation, the HT titer surprisingly increased to 4.21 g/L. Introduction of this operon promoted FADH_2_ regeneration within *E. coli*, playing a crucial role in the hydroxylation of this enzyme.

Shake-flask experiments on pure HT revealed that the pH and DO significantly influenced its stability. It remained relatively stable at pH 6.0 under oxygen-deficient conditions. Because hydroxylation requires oxygen, continuous oxygen restriction is detrimental to HT biosynthesis. Based on these physicochemical properties, a two-stage pH and DO fermentation strategy was developed. During the rapid growth phase, conditions were maintained at pH 6.4 and 20 ± 5% DO. After stabilization, the conditions were adjusted to pH 6.0 and 0%–5% DO. This approach maximized HT retention and facilitated subsequent separation and extraction. Given the ThDP-dependent nature of ARO10 (43), the VB₁ concentration was optimized for HT production, which was determined to be 5 mg/L. This optimal supplementation level ultimately achieved a hydroxytyrosol titer of 9.22 g/L.

Overall, this study optimized the metabolic pathways and fermentation processes to obtain the strain HT32-3 with excellent HT production capacity and provided insights for modifying tyrosine-related metabolites. However, the toxicity of HT requires further investigation. This toxicity may cause growth arrest in later stages of culture, preventing sustained HT production. Identifying the transport proteins that regulate the efflux of tyrosol or HT may be the most direct and effective approach to resolve this issue.

## MATERIALS AND METHODS

### Plasmids, strains, and culture conditions

The plasmids and engineering strains used in this study are listed in the supplemental material. *E. coli* W3110 was used as a starting strain for molecular modification, *E. coli* DH5α was used for plasmid vector construction and cloning, and *E. coli* BL21 was used as a plasmid expression vector. Plasmids pREDCas9 and pGRB were used for the CRISPR-/Cas9-mediated gene editing system. The pET-28a-c(+) vectors carry an N-terminal His Tag/thrombin/T7Tag configuration plus an optional C-terminal His. Plasmid pET-28a was used for the expression of the *ARO10* gene, *ADH6* gene, and *HpaBC* gene.

Ampicillin 100 μg/mL and spectinomycin 50 μg/mL were added when necessary. Isopropyl β-D-thiogalactopyranoside (IPTG) and L-arabinose were added at final concentrations of 0.2 mM and 0.2% (wt/vol), respectively, in the induction experiments.

### Acquisition of target genes

The heterologous genes used in this study were synthetic (Jin Weizhi, Tianjin) and codon-optimized. Endogenous target genes were amplified using the *E. coli* W3110 genome and BL21 genomic DNA as templates. For example, the target gene tktA was obtained by using the *E. coli* W3110 genome as a template and amplified by primer pair yjiK-tktA-s/yjiK-tktA-a. Primers used in this study are provided in the supplemental material.

### Recombinant plasmid construction

The construction of recombinant plasmids was completed by homologous recombination. Taking the construction of the *EcHpaBC* gene overexpression plasmid pET-*EcHpaBC* as an example, first, the primers pET28a-xz-s, pET28a-xz-a, EcHpaBC-s, and EcHpaBC-a were designed using the software CE Design V1.03, and each primer carried the corresponding homologous sequence. Afterward, the primer pairs pET28a-xz-s/pET28a-xz-a and EcHpaBC-s/EcHpaBC-a were used to amplify the linear vector and the target fragment, respectively. Finally, the linear vector was ligated to the target gene fragment by homologous recombination using the ClonExpress II One-Step Cloning Kit (Vazyme Biotech, Nanjing, China).

### Genome modification

The CRISPR/Cas9 gene editing system was utilized in *E. coli* W3110 for gene knockdown and integration. Take knockdown of the *tyrR* gene as an example. First, a pGRB-*tyrR* plasmid was constructed, and a 20 bp spacer region sequence was obtained using CRISPR RGEN Tools screening. Then, a pair of complementary primers (gRNA-tyrR-1 and gRNA-tyrR-2) were synthesized and annealed to form a piece of dsDNA, which contained sequences homologous to the pGRB backbone, and then a linearized pGRB plasmid was connected with the dsDNA and ligated. PCR amplification was performed from *E. coli* W3110 genomic DNA using primers tyrR-up-s/tyrR-up-a and tyrR-dw-s/tyrR-dw-a to obtain the tyrR upstream and downstream homology arms, which were then used as templates for overlapping PCRs using the tyrR-up-s and tyrR-dw-a primers to obtain the tyrR deletion fragment. The fragment was electroporated along the pGRB-*tyrR* plasmid into *E. coli* W3110 receptor cells containing pRED-Cas9, and positive transformants were screened on Luria-Bertani (LB) agar plates containing spectinomycin and ampicillin.

### Shake-flask culture

Seed cultures grown in 5 mL of LB medium (OD_600_ ≥ 2) were inoculated in 500 mL shake flasks containing 27 mL of the sterile medium. They were incubated at 34℃ and 220 rpm for 22 h. The pH (6.4) was adjusted using 25% (vol/vol) ammonia solution and monitored using phenol red. Sterile glucose solution (60%, wt/vol) was supplied when glucose was depleted in the initial culture solution. Xylose 5 g/L was added at once at the beginning of fermentation to induce gene expression driven by the T7 promoter. The medium contained 10.0, 4.0, 1.5, 2.0, 2.0, 2.0, and 4.0 g/L glucose, yeast extract, peptone, citric acid, MgSO_4_⋅7H_2_O, and KH_2_PO_4_, as well as 5, 30, 10, and FeSO_4_⋅7H_2_O, MnSO_4_⋅7H_2_O, and biotin, respectively. Furthermore, the base medium was supplemented with specific substrates for individual experiments: 4 g/L tyrosol, 4 g/L 4-HPP, or 0.2 g/L riboflavin, as required by the respective experimental design. The stability of HT under different pH conditions was assessed by supplementing sterile deionized water with 10 g/L pure HT and adjusting the pH with 25% (vol/vol) ammonia solution. Meanwhile, the effect of DO on HT stability was evaluated in 500 mL shake flasks containing 30 mL of the same HT solution. The flasks were agitated at 30, 150, and 270 rpm to achieve varying DO levels.

### Batch refill fermentation in 5 L fermenters

An appropriate amount of agar slant culture cells was transferred to 3 L of the seed medium in a 5 L bioreactor (Shanghai Baoxing, China). The seed medium and fermentation medium in the bioreactor were the same as those used in shake flasks, except that no phenol red was added. When the OD_600_ reached 12–15, 600 mL of the seed culture was retained, and fresh fermentation medium was added immediately to make the final volume of the fermentation broth 3 L. The whole fermentation process was automatically controlled by adding the ammonia solution (25%, vol/vol) in two stages for pH, 34°C for temperature, and by varying the stirring speed and aeration volume for dissolved oxygen in two stages. Xylose at a final concentration of 5 g/L was added at once at the beginning of fermentation to induce gene expression driven by the T7 promoter. When the substrate glucose was depleted, sterile glucose solution (80%, wt/vol) was replenished appropriately, and the glucose concentration was maintained below 5 g/L. Additionally, the medium was supplemented with 5 mg/L vitamin B₁ as a cofactor.

### Methods of analysis

Absorbance at 600 nm was measured using an ultraviolet/visible spectrophotometer (UV1800, Shanghai Essence Technology Instrument Co., Ltd., Shanghai, China) to detect the cell growth status. A biosensor analyzer (SBA-40E, Shandong Academy of Sciences, Shandong, China) was used for the determination of glucose concentration. Intracellular NAD(P)H and NAD(P)^+^ levels were detected using NADPH/NADP^+^ and NADH/NAD^+^ analysis kits with WST-8 (S0179 and S0175, Beyotime, China). The method for the determination of hydroxytyrosol, 4-HPP, tyrosine, and L-DOPA by highperformance liquid chromatography (HPLC) was as follows: LC-100 liquid chromatograph (UV100 Plus ultraviolet detector); column: Agilent C18 HPLC column (250 mm × 4.6 mm, 5 μm); mobile phase: 5% aqueous acetonitrile phosphate solution; column temperature: 30 ℃; flow rate: 1 mL/min. The ultraviolet detection wavelength was 210 nm. The detection of FADH2 was carried out by HPLC on a Poroshell 120 EC-C18 HPLC column (3 mm × 100 mm, 2.7 μm) with mobile phase A (phosphate buffer, 50 mm, pH 3.1) and mobile phase B (100% acetonitrile). The column temperature was 30℃, the flow rate was 0.8 mL/min, and the ultraviolet detection wavelength was 450 nm.

### Statistical methods

The data in this study represent the mean and standard deviation of three independent experiments. One-way ANOVA and Dunnett’s multiple comparison test were used to determine significant differences between the data. A *P*-value of 0.01 < *P* < 0.05 was considered a significant difference, and a *P*-value of <0.01 was considered a highly significant difference.

## Data Availability

All experimental data and supporting materials in this study are publicly available and can be accessed through the supplemental material or databases. Specific data can be requested by contacting the authors or retrieved from online public databases such as GenBank and KEGG. For any additional data requests, please contact the authors via email.

## SUPPLEMENTAL MATERIAL

Supplemental material aem.02455-25-s0001.docx Tables S1 and S2 and gene sequences.

ASM does not own the copyrights to Supplemental Material that may be linked to, or accessed through, an article. The authors have granted ASM a non-exclusive, world-wide license to publish the Supplemental Material files. Please contact the corresponding author directly for reuse.

## References

[1] Karković Marković A, Torić J, Barbarić M, Jakobušić Brala C. 2019. Hydroxytyrosol, tyrosol and derivatives and their potential effects on human health. Molecules 24:2001. doi:10.3390/molecules24102001 31137753 PMC6571782

[2] López de Las Hazas M-C, Del Saz-Lara A, Cedó L, Crespo MC, Tomé-Carneiro J, Chapado LA, Macià A, Visioli F, Escola-Gil JC, Dávalos A. 2024. Hydroxytyrosol induces dyslipidemia in an ApoB100 humanized mouse model. Mol Nutr Food Res 68:e2300508. doi:10.1002/mnfr.202300508 37933702

[3] Anter J, Quesada-Gómez JM, Dorado G, Casado-Díaz A. 2016. Effect of hydroxytyrosol on human mesenchymal stromal/stem cell differentiation into adipocytes and osteoblasts. Arch Med Res 47:162–171. doi:10.1016/j.arcmed.2016.06.006 27393375

[4] Wen X, Tang S, Wan F, Zhong R, Chen L, Zhang H. 2024. The PI3K/Akt-Nrf2 signaling pathway and mitophagy synergistically mediate hydroxytyrosol to alleviate intestinal oxidative damage. Int J Biol Sci 20:4258–4276. doi:10.7150/ijbs.97263 39247828 PMC11379072

[5] Bertelli M, Kiani AK, Paolacci S, Manara E, Kurti D, Dhuli K, Bushati V, Miertus J, Pangallo D, Baglivo M, Beccari T, Michelini S. 2020. Hydroxytyrosol: a natural compound with promising pharmacological activities. J Biotechnol 309:29–33. doi:10.1016/j.jbiotec.2019.12.016 31884046

[6] Li H, He H, Liu C, Akanji T, Gutkowski J, Li R, Ma H, Wan Y, Wu P, Li D, Seeram NP, Ma H. 2022. Dietary polyphenol oleuropein and its metabolite hydroxytyrosol are moderate skin permeable elastase and collagenase inhibitors with synergistic cellular antioxidant effects in human skin fibroblasts. Int J Food Sci Nutr 73:460–470. doi:10.1080/09637486.2021.1996542 34719319

[7] Gurdo N, Volke DC, McCloskey D, Nikel PI. 2023. Automating the design-build-test-learn cycle towards next-generation bacterial cell factories. N Biotechnol 74:1–15. doi:10.1016/j.nbt.2023.01.002 36736693

[8] Chung D, Kim SY, Ahn J-H. 2017. Production of three phenylethanoids, tyrosol, hydroxytyrosol, and salidroside, using plant genes expressing in Escherichia coli. Sci Rep 7. doi:10.1038/s41598-017-02042-2 PMC5451403 28566694

[9] Li X, Chen Z, Wu Y, Yan Y, Sun X, Yuan Q. 2018. Establishing an artificial pathway for efficient biosynthesis of hydroxytyrosol. ACS Synth Biol 7:647–654. doi:10.1021/acssynbio.7b00385 29281883

[10] Liu H, Wu X, Ma H, Li J, Liu Z, Guo X, Dong J, Zou S, Luo Y. 2022. High-level production of hydroxytyrosol in engineered Saccharomyces cerevisiae ACS Synth Biol 11:3706–3713. doi:10.1021/acssynbio.2c00316 36345886

[11] Wang H, Wang L, Chen J, Hu M, Fang F, Zhou J. 2023. Promoting FADH2 regeneration of hydroxylation for high-level production of hydroxytyrosol from glycerol in Escherichia coli. J Agric Food Chem 71:16681–16690. doi:10.1021/acs.jafc.3c05477 37877749

[12] Xiong T, Li X, Liu W, Yue H, Liu J, Bai D, Li W, Fan G. 2025. Multienzyme cascade for synthesis of hydroxytyrosol via engineered Escherichia coli. Sci Rep 15:471. doi:10.1038/s41598-024-84624-5 39748076 PMC11696566

[13] Orsi E, Claassens NJ, Nikel PI, Lindner SN. 2022. Optimizing microbial networks through metabolic bypasses. Biotechnol Adv 60:108035. doi:10.1016/j.biotechadv.2022.108035 36096403

[14] Xia Y, Qi L, Shi X, Chen K, Peplowski L, Chen X. 2023. Construction of an Escherichia coli cell factory for de novo synthesis of tyrosol through semi-rational design based on phenylpyruvate decarboxylase ARO10 engineering. Int J Biol Macromol 253:127385. doi:10.1016/j.ijbiomac.2023.127385 37848109

[15] Chen W, Yao J, Meng J, Han W, Tao Y, Chen Y, Guo Y, Shi G, He Y, Jin J-M, Tang S-Y. 2019. Promiscuous enzymatic activity-aided multiple-pathway network design for metabolic flux rearrangement in hydroxytyrosol biosynthesis. Nat Commun 10:960. doi:10.1038/s41467-019-08781-2 30814511 PMC6393456

[16] Bai Y, Bi H, Zhuang Y, Liu C, Cai T, Liu X, Zhang X, Liu T, Ma Y. 2014. Production of salidroside in metabolically engineered Escherichia coli. Sci Rep 4. doi:10.1038/srep06640 PMC4200411 25323006

[17] Nakashima N, Tamura T. 2012. A new carbon catabolite repression mutation of Escherichia coli, mlc∗, and its use for producing isobutanol. J Biosci Bioeng 114:38–44. doi:10.1016/j.jbiosc.2012.02.029 22561880

[18] Nakashima N, Akita H, Hoshino T. 2014. Establishment of a novel gene expression method, BICES (biomass-inducible chromosome-based expression system), and its application to the production of 2,3-butanediol and acetoin. Metab Eng 25:204–214. doi:10.1016/j.ymben.2014.07.011 25108217

[19] Bushin LB, Alter TB, Alván-Vargas MVG, Dürr L, Olson EC, Avila MJ, Volke DC, Puiggené Ò, Kim T, Deravi LF, Feist AM, Nikel PI, Moore BS. 2025. Growth-coupled microbial biosynthesis of the animal pigment xanthommatin. Nat Biotechnol. doi:10.1038/s41587-025-02867-7 PMC13571322 41184490

[20] Bilal M, Wang S, Iqbal HMN, Zhao Y, Hu H, Wang W, Zhang X. 2018. Metabolic engineering strategies for enhanced shikimate biosynthesis: current scenario and future developments. Appl Microbiol Biotechnol 102:7759–7773. doi:10.1007/s00253-018-9222-z 30014168

[21] Zhang X, Wang X, Shanmugam KT, Ingram LO. 2011. L-malate production by metabolically engineered Escherichia coli. Appl Environ Microbiol 77:427–434. doi:10.1128/AEM.01971-10 21097588 PMC3020529

[22] Siddiquee KAZ, Arauzo-Bravo MJ, Shimizu K. 2004. Effect of a pyruvate kinase (pykF-gene) knockout mutation on the control of gene expression and metabolic fluxes in Escherichia coli. FEMS Microbiol Lett 235:25–33. doi:10.1016/j.femsle.2004.04.004 15158258

[23] Bongaerts J, Krämer M, Müller U, Raeven L, Wubbolts M. 2001. Metabolic engineering for microbial production of aromatic amino acids and derived compounds. Metab Eng 3:289–300. doi:10.1006/mben.2001.0196 11676565

[24] Kikuchi Y, Tsujimoto K, Kurahashi O. 1997. Mutational analysis of the feedback sites of phenylalanine-sensitive 3-deoxy-D-arabino-heptulosonate-7-phosphate synthase of Escherichia coli. Appl Environ Microbiol 63:761–762. doi:10.1128/aem.63.2.761-762.1997 9023954 PMC168366

[25] Hudson GS, Howlett GJ, Davidson BE. 1983. The binding of tyrosine and NAD+ to chorismate mutase/prephenate dehydrogenase from Escherichia coli K12 and the effects of these ligands on the activity and self-association of the enzyme. Analysis in terms of a model. J Biol Chem 258:3114–3120. 6338013

[26] Christopherson RI. 1985. Chorismate mutase-prephenate dehydrogenase from Escherichia coli: cooperative effects and inhibition by L-tyrosine. Arch Biochem Biophys 240:646–654. doi:10.1016/0003-9861(85)90072-4 3896148

[27] Pittard J, Camakaris H, Yang J. 2005. The TyrR regulon. Mol Microbiol 55:16–26. doi:10.1111/j.1365-2958.2004.04385.x 15612913

[28] Chao YP, Lai ZJ, Chen P, Chern JT. 1999. Enhanced conversion rate of L-phenylalanine by coupling reactions of aminotransferases and phosphoenolpyruvate carboxykinase in Escherichia coli K-12. Biotechnol Prog 15:453–458. doi:10.1021/bp990044f 10356262

[29] Sivaraman J, Li Y, Larocque R, Schrag JD, Cygler M, Matte A. 2001. Crystal structure of histidinol phosphate aminotransferase (HisC) from Escherichia coli, and its covalent complex with pyridoxal-5’-phosphate and l-histidinol phosphate. J Mol Biol 311:761–776. doi:10.1006/jmbi.2001.4882 11518529

[30] Lee KH, Park JH, Kim TY, Kim HU, Lee SY. 2007. Systems metabolic engineering of Escherichia coli for L-threonine production. Mol Syst Biol 3:149. doi:10.1038/msb4100196 18059444 PMC2174629

[31] Liu F, Qimuge S, Hao J, Yan H, Bach T, Fan L, Morigen S. 2014. AspC-mediated aspartate metabolism coordinates the Escherichia coli cell cycle. PLoS One 9:e92229. doi:10.1371/journal.pone.0092229 24670900 PMC3966765

[32] Sun W, Ding D, Bai D, Lin Y, Zhu Y, Zhang C, Zhang D. 2023. Transcriptomics and metabolomics analysis of L-phenylalanine overproduction in Escherichia coli. Microb Cell Fact 22:65. doi:10.1186/s12934-023-02070-w 37024921 PMC10080781

[33] Trantas E, Navakoudis E, Pavlidis T, Nikou T, Halabalaki M, Skaltsounis L, Ververidis F. 2019. Dual pathway for metabolic engineering of Escherichia coli to produce the highly valuable hydroxytyrosol. PLoS One 14:e0212243. doi:10.1371/journal.pone.0212243 31682615 PMC6828502

[34] Lee ME, DeLoache WC, Cervantes B, Dueber JE. 2015. A highly characterized yeast toolkit for modular, multipart assembly. ACS Synth Biol 4:975–986. doi:10.1021/sb500366v 25871405

[35] Fedorec AJH, Ozdemir T, Doshi A, Ho Y-K, Rosa L, Rutter J, Velazquez O, Pinheiro VB, Danino T, Barnes CP. 2019. Two new plasmid post-segregational killing mechanisms for the implementation of synthetic gene networks in Escherichia coli. iScience 14:323–334. doi:10.1016/j.isci.2019.03.019 30954530 PMC6489366

[36] Fernández-Cabezón L, Rosich i Bosch B, Kozaeva E, Gurdo N, Nikel PI. 2022. Dynamic flux regulation for high-titer anthranilate production by plasmid-free, conditionally-auxotrophic strains of Pseudomonas putida. Metab Eng 73:11–25. doi:10.1016/j.ymben.2022.05.008 35659519

[37] Yu X, Lv H, Luo H, Zhu X, Wu J, Zhang K. 2025. High level food-grade expression of maltogenic amylase in Bacillus subtilis through genomic integration and comA overexpression. Int J Biol Macromol 309:143060. doi:10.1016/j.ijbiomac.2025.143060 40220825

[38] Snoeck S, Guidi C, De Mey M. 2024. “Metabolic burden” explained: stress symptoms and its related responses induced by (over)expression of (heterologous) proteins in Escherichia coli. Microb Cell Fact 23:96. doi:10.1186/s12934-024-02370-9 38555441 PMC10981312

[39] Smith JJ, Lilly MD, Fox RI. 1990. The effect of agitation on the morphology and penicillin production of Penicillium chrysogenum. Biotechnol Bioeng 35:1011–1023. doi:10.1002/bit.260351009 18588247

[40] Yuan J-J, Qin FGF, Tu J-L, Li B. 2017. Preparation, characterization, and antioxidant activity evaluation of liposomes containing water-soluble hydroxytyrosol from Olive. Molecules 22:870. doi:10.3390/molecules22060870 28538693 PMC6152771

[41] Ma L, Chen Z, Li X, Liu W, Yu Z, Li C, Gong Y, Xu Q. 2025. De novo synthesis of tyramine in engineered Escherichia coli using two-stage dissolved oxygen-controlled fermentation. J Agric Food Chem 73:4174–4184. doi:10.1021/acs.jafc.4c11385 39905769

[42] Duggleby RG. 2006. Domain relationships in thiamine diphosphate-dependent enzymes. Acc Chem Res 39:550–557. doi:10.1021/ar068022z 16906751

[43] Kneen MM, Stan R, Yep A, Tyler RP, Saehuan C, McLeish MJ. 2011. Characterization of a thiamin diphosphate-dependent phenylpyruvate decarboxylase from Saccharomyces cerevisiae. FEBS J 278:1842–1853. doi:10.1111/j.1742-4658.2011.08103.x 21501384

[44] Versées W, Spaepen S, Wood MDH, Leeper FJ, Vanderleyden J, Steyaert J. 2007. Molecular mechanism of allosteric substrate activation in a thiamine diphosphate-dependent decarboxylase. J Biol Chem 282:35269–35278. doi:10.1074/jbc.M706048200 17905741

[45] Deng L, Ping J, Liu Z, Linghu K, Zhang H, Shan X, Zeng W, Li J, Zhou J. 2026. Efficient synthesis of l-DOPA in Escherichia coli via cofactor and enzyme engineering. Synth Syst Biotechnol 11:226–236. doi:10.1016/j.synbio.2025.09.011 41104292 PMC12523793

[46] Kozaeva E, Volkova S, Matos MRA, Mezzina MP, Wulff T, Volke DC, Nielsen LK, Nikel PI. 2021. Model-guided dynamic control of essential metabolic nodes boosts acetyl-coenzyme A-dependent bioproduction in rewired Pseudomonas putida. Metab Eng 67:373–386. doi:10.1016/j.ymben.2021.07.014 34343699

[47] Qi L, Liu C, Peplowski L, Shen W, Yang H, Xia Y, Chen X. 2023. Efficient production of hydroxytyrosol by directed evolution of HpaB in Escherichia coli. Biochem Biophys Res Commun 663:16–24. doi:10.1016/j.bbrc.2023.04.024 37116393

[48] Sotomayor-Vivas C, Hernández-Lemus E, Dorantes-Gilardi R. 2022. Linking protein structural and functional change to mutation using amino acid networks. PLoS One 17:e0261829. doi:10.1371/journal.pone.0261829 35061689 PMC8782487

[49] Liu L, Li W, Li X, Sun X, Yuan Q. 2019. Constructing an efficient salicylate biosynthesis platform by Escherichia coli chromosome integration. J Biotechnol 298:5–10. doi:10.1016/j.jbiotec.2019.04.004 30959138

[50] Meng J, Wang B, Liu D, Chen T, Wang Z, Zhao X. 2016. High-yield anaerobic succinate production by strategically regulating multiple metabolic pathways based on stoichiometric maximum in Escherichia coli. Microb Cell Fact 15:141. doi:10.1186/s12934-016-0536-1 27520031 PMC4983090

[51] Koma D, Kishida T, Yoshida E, Ohashi H, Yamanaka H, Moriyoshi K, Nagamori E, Ohmoto T. 2020. Chromosome engineering to generate plasmid-free phenylalanine- and tyrosine-overproducing Escherichia coli strains that can be applied in the generation of aromatic-compound-producing bacteria. Appl Environ Microbiol 86:e00525-20. doi:10.1128/AEM.00525-20 32414798 PMC7357484

[52] Gowrishankar J, Pittard J. 1982. Regulation of phenylalanine biosynthesis in Escherichia coli K-12: control of transcription of the pheA operon. J Bacteriol 150:1130–1137. doi:10.1128/jb.150.3.1130-1137.1982 7042684 PMC216333

[53] Xue Y, Chen X, Yang C, Chang J, Shen W, Fan Y. 2017. Engineering Eschericha coli for enhanced tyrosol production. J Agric Food Chem 65:4708–4714. doi:10.1021/acs.jafc.7b01369 28530096

