# Supplementary material for: Systems metabolic engineering for hydroxytyrosol production in Escherichia coli
Source: Appl Environ Microbiol. 2026 Apr 17;92(5):e02455-25. doi: 10.1128/aem.02455-25 (PMC13188884; doi:10.1128/aem.02455-25)
Supplement: Supplemental material — Tables S1 and S2 and gene sequences. [file aem.02455-25-s0001.docx]

1. The plasmids, engineered strains and primer sequences used in this study are listed in Tables 1 and 2, respectively.

Table 1 Strains and plasmids used in this study.

| **Strains** | **Characteristics** | **Source** |
| --- | --- | --- |
| *E. coli* DH5α | Host for cloning | Lab stock |
| *E. coli* W3110 | Wild type, starting strain | Lab stock |
| *E. coli* BL21 | Wild type, starting strain | Lab stock |
| HT00 | *E. coli* W3110, ∆*lacI*::PxylF-*T7RNAP* | This study |
| HT01 | BL21, pET-28a | This study |
| HT02 | BL21, pET-*EcHpaBC* | This study |
| HT03 | BL21, pET-*KpHpaBC* | This study |
| HT04 | BL21, pET-*PaHpaBC* | This study |
| HT05 | HT00, pET-*ARO10-ADH6* | This study |
| HT06 | HT00, pET-*ARO10^D331V^-ADH6* | This study |
| HT07 | HT00, pET-*ARO10^D331C^-ADH6* | This study |
| HT08 | HT00, pET-*ARO10^D331S^-ADH6* | This study |
| HT09 | HT00, pET-*ARO10-ADH6-EcHpaBC* | This study |
| HT10 | HT09, ∆*PykF* | This study |
| HT11 | HT10, *yjiK*::Ptrc-*tktA*, *yjiP*::Ptrc-*talB* | This study |
| HT12 | HT11, *yeeL*::Ptrc-*aroG^D146N^* | This study |
| HT13 | HT12, *yeeP*::Ptrc-*tyrA^M53I/A354V^* | This study |
| HT14 | HT13, ∆*tyrR* | This study |
| HT15 | HT14, ∆*tyrB* | This study |
| HT16 | HT14, ∆*hisC* | This study |
| HT17 | HT14, ∆*aspC* | This study |
| HT18 | HT15, ∆*pheA*, ∆*feaB* | This study |
| HT19 | HT18, *ycdN*::Ptrc-*ARO10* | This study |
| HT20 | HT18, *ycjV*::Ptrc-*ADH6* | This study |
| HT21 | HT18, *molR*::Ptrc-*EcHpaBC* | This study |
| HT22 | HT18, *yghX*::Ptrc-*ARO10-ADH6* | This study |
| HT23 | HT18, *ydeU*::Ptrc-*ARO10-ADH6-EcHpaBC* | This study |
| HT24 | HT22, *mbhA*::Ptrc-*ARO10-ADH6* | This study |
| HT25 | HT24, *rph*::Ptrc-*ARO10-ADH6* | This study |
| HT26 | HT25, *yciQ*::Ptrc-*ARO10-ADH6* | This study |
| HT27 | HT26, *gapC*::Ptrc-*ARO10-ADH6* | This study |
| HT28 | HT27, *yfaS*::Ptrc-*ARO10-ADH6* | This study |
| HT29 | HT27, *pntAB*::Ptrc-*pntAB* | This study |
| HT30 | HT29, *ycgH*::Ptrc-*pntAB* | This study |
| HT31 | HT30, *insB*::Ptrc-*pntAB* | This study |
| HT32 | HT30, *ilvG*::Ptrc-*ribA-ribH-ribE-ribC* | This study |
| HT32-1 | HT30, *ygaY*::Pj23105::*ribA-ribH-ribE-ribC* | This study |
| HT32-2 | HT30, *ylbE*::Pj23101::*ribA-ribH-ribE-ribC* | This study |
| HT32-3 | HT30, *yjgX*::Pj23100::*ribA-ribH-ribE-ribC* | This study |
| **Plasmid** | **Characteristics** | **Source** |
| pGRB | gRNA expression vector | Lab stock |
| pRed-cas9 | Cas9 expression vector | Lab stock |
| pET-28a | Gene expression vector | Lab stock |
| pET-*EcHpaBC* | containing *EcHpaBC*; KanR | This study |
| pET-*KpHpaBC* | containing *KpHpaBC*; KanR | This study |
| pET-*PaHpaBC* | containing *PaHpaBC*; KanR | This study |
| pET-*ARO10-ADH6* | containing *ARO10* and *ADH6*; KanR | This study |
| pET-*ARO10^D331C^-ADH6* | containing *ARO10 ^D331C^* and *ADH6*; KanR | This study |
| pET-*ARO10^D331V^-ADH6* | containing *ARO10 ^D331V^* and *ADH6*; KanR | This study |
| pET-*ARO10^D331S^-ADH6* | containing *ARO10 ^D331S^* and *ADH6*; KanR | This study |
| pET-*ARO10-ADH6-EcHpaBC* | containing *ARO10* and *ADH6* and *EcHpaBC*; KanR | This study |
| pGRB-*lacI* | used for transcribing sgRNA targeting *lacI* | This study |
| pGRB-*PykF* | used for transcribing sgRNA targeting *PykF* | This study |
| pGRB-*tyrR* | used for transcribing sgRNA targeting *tyrR* | This study |
| pGRB-*tyrB* | used for transcribing sgRNA targeting *tyrB* | This study |
| pGRB-*hisC* | used for transcribing sgRNA targeting *hisC* | This study |
| pGRB-*aspC* | used for transcribing sgRNA targeting *aspC* | This study |
| pGRB-*pheA* | used for transcribing sgRNA targeting *pheA* | This study |
| pGRB-*feaB* | used for transcribing sgRNA targeting *feaB* | This study |
| pGRB-*yjiK* | used for transcribing sgRNA targeting *yjiK* | This study |
| pGRB-*yjiP* | used for transcribing sgRNA targeting *yjiP* | This study |
| pGRB-*yeeL* | used for transcribing sgRNA targeting *yeeL* | This study |
| pGRB-*yeeP* | used for transcribing sgRNA targeting *yeeP* | This study |
| pGRB-*ycdN* | used for transcribing sgRNA targeting *ycdN* | This study |
| pGRB-*ycjV* | used for transcribing sgRNA targeting *ycjV* | This study |
| pGRB-*molR* | used for transcribing sgRNA targeting *molR* | This study |
| pGRB-*yghX* | used for transcribing sgRNA targeting *yghX* | This study |
| pGRB-*ydeU* | used for transcribing sgRNA targeting *ydeU* | This study |
| pGRB-*mbhA* | used for transcribing sgRNA targeting *mbhA* | This study |
| pGRB-*rph* | used for transcribing sgRNA targeting *rph* | This study |
| pGRB-*gapC* | used for transcribing sgRNA targeting *gapC* | This study |
| pGRB-*yfaS* | used for transcribing sgRNA targeting *yfaS* | This study |
| pGRB-*pntAB* | used for transcribing sgRNA targeting *pntAB* | This study |
| pGRB-*ycgH* | used for transcribing sgRNA targeting *ycgH* | This study |
| pGRB-*insB* | used for transcribing sgRNA targeting *insB* | This study |
| pGRB-*ilvG* | used for transcribing sgRNA targeting *ilvG* | This study |
| pGRB-*ygaY* | used for transcribing sgRNA targeting *ygaY* | This study |
| pGRB-*ylbE* | used for transcribing sgRNA targeting *ylbE* | This study |
| pGRB-*yjgX* | used for transcribing sgRNA targeting *yjgX* | This study |

Table 2 Primers used in this study.

| **Primers** | **Sequence (5' end to 3' end)** |
| --- | --- |
| pET28a-xz-s | CTAGCATAACCCCTTGGGGC |
| pET28a-xz-a | GGAATTGTTATCCGCTCACAATTCCCCTATAGTGAGTCGTATTAGCGCAACGCAATTAATGTAAGTTAGC |
| *EcHpaBC*-s | TGAGCGGATAACAATTCCCCTCTAGAAATAATTTTGTTTAACTTTAAGAAGGAGATATACCATGAAACCAGAAGATTTCCGCGC |
| *EcHpaBC*-a | GCCCCAAGGGGTTATGCTAGTTAAATCGCAGCTTCCATTTCCAGC |
| *KpHpaBC*-s | TGAGCGGATAACAATTCCCCTCTAGAAATAATTTTGTTTAACTTTAAGAAGGAGATATACCATGAAACCGGAAGATTTTCGCG |
| *KpHpaBC*-a | GCCCCAAGGGGTTATGCTAGTTACACCGCCACTTCCATTTCCA |
| *PaHpaBC*-s | ACCTCACGCAAAATAACACAGTCAAATCAATCAAAATGAAGCCAGAAGACTTCAGAGCT |
| *PaHpaBC*-a | TGATCTATCGATTTCAATTCAATTCAATTTAAGCAGCTCTTCTTGGACATTGCAATCT |
| pET-*ARO10*-s | CAGATAAAACGAAAGGCCCAGTCTTTCGACTGAGCCTTTCGTTTTATTTGTTACTGGCGATTGTCATTCG |
| pET-*ARO10*-a | TTTCAATCTCCTATTATTTCCTTAGAAGCTTATTTTTTGTTGCGTTTCAGCGCG |
| pET- *ARO10^D331C^*-s | CAGATAAAACGAAAGGCCCAGTCTTTCGACTGAGCCTTTCGTTTTATTTGTTACTGGCGATTGTCATTCG |
| pET- *ARO10^D331C^*-a | TTTCAATCTCCTATTATTTCCTTAGAAGCTTATTTTTTGTTGCGTTTCAGCGCG |
| pET- *ARO10^D331V^*-s | CAGATAAAACGAAAGGCCCAGTCTTTCGACTGAGCCTTTCGTTTTATTTGTTACTGGCGATTGTCATTCG |
| pET- *ARO10^D331V^*-a | TTTCAATCTCCTATTATTTCCTTAGAAGCTTATTTTTTGTTGCGTTTCAGCGCG |
| pET- *ARO10^D331S^*-s | CAGATAAAACGAAAGGCCCAGTCTTTCGACTGAGCCTTTCGTTTTATTTGTTACTGGCGATTGTCATTCG |
| pET- *ARO10^D331S^*-a | TTTCAATCTCCTATTATTTCCTTAGAAGCTTATTTTTTGTTGCGTTTCAGCGCG |
| pET-*ADH6*-s | TGAAACGCAACAAAAAATAAGCTTCTAAGGAAATAATAGGAGATTGAAAATGAGCTATCCGGAAAAATTTGAAGGC |
| pET-*ADH6*-a | TTAATCGCTAAATTCTTTATCATAGCCCACCAG |
| pET-*EcHpaBC*-s | CTGGTGGGCTATGATAAAGAATTTAGCGATTAAAGGAAACAGTATTCATGATGAAACCGGAAGATTTTCGCG |
| pET-*EcHpaBC*-a | GCCCCAAGGGGTTATGCTAGTTACACCGCCACTTCCATTTCCA |
| pGRB-*lacI*-s | AGTCCTAGGTATAATACTAGTGCCACGTTTCTGCGAAAACGGTTTTAGAGCTAGAA |
| pGRB-*lacI*-a | TTCTAGCTCTAAAACCGTTTTCGCAGAAACGTGGCACTAGTATTATACCTAGGACT |
| *lacI*-up-s | GACTATCAACTGGCACGGGAA |
| *lacI*-up-a | CGATTTGCTGGTGACCCAAGCGGGAAACGGTCTGATAAG |
| *lacI*-dw-s | CTTATCAGACCGTTTCCCGCTTGGGTCACCAGCAAATCG |
| *lacI*-dw-a | TTTTCACCAGTGAGACGGGC |
| *T7RNAP*-s | TCGCGGTGATTGTTACTTATTAAAACTGTCCTCTAACTACAGAAGGCCCTACACCATGGGATTTACTAACTGGAAGAGGC |
| *T7RNAP*-a | GCGCACGAAAAGCATCAGGTCTTTCCTTCGAAGGGGATCCGGAGTCGTATTGATTTGGCGTTACGCGAACGCGAAGTCCG |
| pGRB-*PykF*-s | AGTCCTAGGTATAATACTAGTGACGGCATCATGGTTGCGCGGTTTTAGAGCTAGAA |
| pGRB-*PykF*-a | TTCTAGCTCTAAAACCGCGCAACCATGATGCCGTCACTAGTATTATACCTAGGACT |
| *PykF*-up-s | GTCTGATCGGTATGGAAGT |
| *PykF*-up-a | GAGTCGGGCGTGGGTTTTTGGAGATGATGTGGATGTTT |
| *PykF*-dw-s | AAACATCCACATCATCTCCAAAAACCCACGCCCGACTC |
| *PykF*-dw-a | GGCACAACGCCTTTGCTCA |
| pGRB-*yjiK*-s | AGTCCTAGGTATAATACTAGTTACGGGGTTTCTCCGCGTTAGTTTTAGAGCTAGAA |
| pGRB-*yjiK*-a | TTCTAGCTCTAAAACTAACGCGGAGAAACCCCGTAACTAGTATTATACCTAGGACT |
| *yjiK*-up-s | GAATCTCACCTGCGAATGCC |
| *yjiK*-up-a | TGTGTGAAATTGTTATCCGCTCACAATTCCACACATTATACGAGCCGGATGATTAATTGTCAAACACCATCCTGCTGGCAATAAA |
| *yjiK*-dw-s | AAAGACTGGGCCTTTCGTTTTATCTGTTGTTTGTCGGTGAACGCTCTCCTGAGTAGGACAAATTTGCGATGGATGCCTCTGGT |
| *yjiK*-dw-a | CCTAAGGTCAGAACCAGCATC |
| *yjiK*-*tktA*-s | TCGTATAATGTGTGGAATTGTGAGCGGATAACAATTTCACACAGGAAACAGACCATGTCCTCACGTAAAGAGCTTGC |
| *yjiK*-*tktA*-a | AAACAACAGATAAAACGAAAGGCCCAGTCTTTCGACTGAGCCTTTCGTTTTATTTGTTACAGCAGTTCTTTTGCTTTCGCAA |
| pGRB-*yjiP*-s | AGTCCTAGGTATAATACTAGTTGGTCGGTAAAGACCCGCGAGTTTTAGAGCTAGAA |
| pGRB-*yjiP*-a | TTCTAGCTCTAAAACTCGCGGGTCTTTACCGACCAACTAGTATTATACCTAGGACT |
| *yjiP*-up-s | GCCATACCGCCAGCAAGAT |
| *yjiP*-up-a | AATTGTTATCCGCTCACAATTCCACACATTATACGAGCCGGATGATTAATTGTCAAGCAGATATTCCCCTTTCCACC |
| *yjiP*-dw-s | AAAGACTGGGCCTTTCGTTTTATCTGTTGTTTGTCGGTGAACGCTCTCCTGAGTAGGACAAATGACGGATGACAAACGCAAAGC |
| *yjiP*-dw-a | AAAGGCGGATTTTTACTGTGGA |
| *yjiP-talB*-s | CTCGTATAATGTGTGGAATTGTGAGCGGATAACAATTTCACACAGGAAACAGACCATGACGGACAAATTGACCTCCCT |
| *yjiP-talB*-a | GATAAAACGAAAGGCCCAGTCTTTCGACTGAGCCTTTCGTTTTATTTGTTACAGCAGATCGCCGATCATTTTTTC |
| pGRB-*yeeL*-s | AGTCCTAGGTATAATACTAGTAACACAGCAATACGGTACGCGTTTTAGAGCTAGAA |
| pGRB-*yeeL*-a | TTCTAGCTCTAAAACGCGTACCGTATTGCTGTGTTACTAGTATTATACCTAGGACT |
| *yeeL*-up-s | TTCATCGGGACGAGTGGAGA |
| *yeeL*-up-a | AATTGTTATCCGCTCACAATTCCACACATTATACGAGCCGGATGATTAATTGTCAACCATAGCATCGCCAATCTGA |
| *yeeL*-dw-s | AAAGACTGGGCCTTTCGTTTTATCTGTTGTTTGTCGGTGAACGCTCTCCTGAGTAGGACAAATACCCAAAGGTGAAGATAAAGCC |
| *yeeL*-dw-a | CATTCCCTCTACAGAACTAGCCCTT |
| *yeeL-aroG^D146N^*-s | TCCGGCTCGTATAATGTGTGGAATTGTGAGCGGATAACAATTTCACACAGGAAACAGACCATGAATTATCAGAACGACGATTTACG |
| *yeeL-aroG^D146N^*-a | CACCGACAAACAACAGATAAAACGAAAGGCCCAGTCTTTCGACTGAGCCTTTCGTTTTATTTGTTACCCGCGACGCGCTTT |
| pGRB-*yeeP*-s | AGTCCTAGGTATAATACTAGTAGGCGGTATTCCGTCTGTTCGTTTTAGAGCTAGAA |
| pGRB-*yeeP*-a | TTCTAGCTCTAAAACGAACAGACGGAATACCGCCTACTAGTATTATACCTAGGACT |
| *yeeP*-up-s | GGTCAGGAGGTAACTTATCAGCG |
| *yeeP*-up-a | AATTGTTATCCGCTCACAATTCCACACATTATACGAGCCGGATGATTAATTGTCAAATGGCAGGGCTCCGTTTT |
| *yeeP*-dw-s | AAAGACTGGGCCTTTCGTTTTATCTGTTGTTTGTCGGTGAACGCTCTCCTGAGTAGGACAAATGAACTGGATTTTCTTCTGAACCTGT |
| *yeeP*-dw-a | ACGATGTCAGCAGCCAGCA |
| *yeeP-tyrA^M53I/A354V^*-s | AATGTGTGGAATTGTGAGCGGATAACAATTTCACACAGGAAACAGACCATGGTTGCTGAATTGACCGCAT |
| *yeeP-tyrA^M53I/A354V^*-a | CAGATAAAACGAAAGGCCCAGTCTTTCGACTGAGCCTTTCGTTTTATTTGTTACTGGCGATTGTCATTCG |
| pGRB-*tyrR*-s | AGTCCTAGGTATAATACTAGTACACGTCCTGACCGGTGCGGGTTTTAGAGCTAGAA |
| pGRB-*tyrR*-a | TTCTAGCTCTAAAACCCGCACCGGTCAGGACGTGTACTAGTATTATACCTAGGACT |
| *tyrR*-up-s | CGCCGTATTGCGGGTGTTA |
| *tyrR*-up-a | CGCTCTGGGGCTTGCCTGATCTTGCGGTTCGCTTTCCA |
| *tyrR*-dw-s | AGTCCTAGGTATAATACTAGTACACGTCCTGACCGGTGCGGGTTTTAGAGCTAGAA |
| *tyrR*-dw-a | TTCTAGCTCTAAAACCCGCACCGGTCAGGACGTGTACTAGTATTATACCTAGGACT |
| pGRB-*tyrB*-s | CGCCGTATTGCGGGTGTTA |
| pGRB-*tyrB*-a | CGCTCTGGGGCTTGCCTGATCTTGCGGTTCGCTTTCCA |
| *tyrB*-up-s | TGGAAAGCGAACCGCAAGATCAGGCAAGCCCCAGAGCG |
| *tyrB*-up-a | GCCAACCCGACGGAAAGTG |
| *tyrB*-dw-s | AGTCCTAGGTATAATACTAGTCCATGTTGCCACAACCCAACGTTTTAGAGCTAGAA |
| *tyrB*-dw-a | TTCTAGCTCTAAAACGTTGGGTTGTGGCAACATGGACTAGTATTATACCTAGGACT |
| pGRB-*hisC*-s | GCTGGAGTTAACCCGCGGTAGTTTTAGAGCTAGAAATAGCAAGTTAA |
| pGRB-*hisC*-a | TACCGCGGGTTAACTCCAGCACTAGTATTATACCTAGGACTGAGC |
| *hisC*-up-s | ATGAGCACCGTGACTATTACCGATT |
| *hisC*-up-a | GATCCCACAAAGATTTAAACACCGCAGAGTAAGCTGAAACTCCACGGCA |
| *hisC*-dw-s | TGCCGTGGAGTTTCAGCTTACTCTGCGGTGTTTAAATCTTTGTGGGATC |
| *hisC*-dw-a | TCAAACTTGCTCCGCACGTAAG |
| pGRB-*aspC*-s | AAGAGCGTCTTTAACTCTGCGTTTTAGAGCTAGAAATAGCAAGTTAA |
| pGRB-*aspC*-a | GCAGAGTTAAAGACGCTCTTACTAGTATTATACCTAGGACTGAGC |
| *aspC*-up-s | ATGTTTGAGAACATTACCGCCGC |
| *aspC*-up-a | TCAGGCCACTGAAGGAGAACATGTTCGAGCAGATACTGTTCAGCCT |
| *aspC*-dw-s | AGGCTGAACAGTATCTGCTCGAACATGTTCTCCTTCAGTGGCCTGA |
| *aspC*-dw-a | TTACAGCACTGCCACAATCGC |
| pGRB-*pheA*-s | CGGCACTTTGTACGGTTTGCGTTTTAGAGCTAGAAATAGCAAGTTAA |
| pGRB-*pheA*-a | GCAAACCGTACAAAGTGCCGACTAGTATTATACCTAGGACTGAGC |
| *pheA*-up-s | AAACATGAAACACATACCGTTTTTCTTCGC |
| *pheA*-up-a | CCCCTAACTCTTTCAATGCTTTTTGCATTAGTTTGGCTTTTCCCACCTCGA |
| *pheA*-dw-s | TCGAGGTGGGAAAAGCCAAACTAATGCAAAAAGCATTGAAAGAGTTAGGGG |
| *pheA*-dw-a | TTTATTGACAGTTTCCGCAAGGTGG |
| pGRB-*feaB*-s | AGTCCTAGGTATAATACTAGTGATCCGCAATGGGTTATTGAGTTTTAGAGCTAGAA |
| pGRB-*feaB*-a | TTCTAGCTCTAAAACTCAATAACCCATTGCGGATCACTAGTATTATACCTAGGACT |
| *feaB*-up-s | ATGACAGAGCCGCATGTAGC |
| *feaB*-up-a | TTCACGCTCTGCGGGTAATCG |
| *feaB*-dw-s | CGCGTCGCTGGGCCGGGCGATTACCCGCAGAGCGTGAAAGAAGAGGCGTTACAACTGGCA |
| *feaB*-dw-a | TTAATACCGTACACACACCGACTTAGTTT |
| pGRB-*ycdN*-s | AGTCCTAGGTATAATACTAGTGCGTGGAAATCATCATGGCTGTTTTAGAGCTAGAA |
| pGRB-*ycdN*-a | TTCTAGCTCTAAAACAGCCATGATGATTTCCACGCACTAGTATTATACCTAGGACT |
| *ycdN*-up-s | GATTTTGACGCCACCAACACC |
| *ycdN*-up-a | GTTATCCGCTCACAATTCCACACATTATACGAGCCGGATGATTAATTGTCAACCAATCCACATCACACAATCCATC |
| *ycdN*-dw-s | CTGGGCCTTTCGTTTTATCTGTTGTTTGTCGGTGAACGCTCTCCTGAGTAGGACAAATGAAGGGATTTTTGGCTATCAGG |
| *ycdN*-dw-a | CATATCGTATTCGCCAGGCTG |
| *ycdN*-*ARO10*-s | TGTGTGGAATTGTGAGCGGATAACAATTTCACACAGGAAACAGACCATGGCGCCGGTGACCATT |
| *ycdN*-*ARO10*-a | ACGAAAGGCCCAGTCTTTCGACTGAGCCTTTCGTTTTATTTGTTATTTTTTGTTGCGTTTCAGCGCG |
| pGRB-*ycjV*-s | AGTCCTAGGTATAATACTAGTCAAAGCACGCAATATAGCGAGTTTTAGAGCTAGAA |
| pGRB-*ycjV*-a | TTCTAGCTCTAAAACTCGCTATATTGCGTGCTTTGACTAGTATTATACCTAGGACT |
| *ycjV*-up-s | GGAAATCTTTCTCGCCGCCT |
| *ycjV*-up-a | TATCCGCTCACAATTCCACACAACATACGAGCCGGAAGCATAAAGTGTAAACGCCGCTGATCTCCTCAAG |
| *ycjV*-dw-s | AAAGACTGGGCCTTTCGTTTTATCTGTTGTTTGTCGGTGAACGCTCTCCTGAGTAGGACAAATGCATAAGCCCATCGTGATGGG |
| *ycjV*-dw-a | CTTCGCCATAACCCTCGACG |
| *ycjV-ADH6*-s | CATCCGGCTCGTATAATGTGTGGAATTGTGAGCGGATAACAATTTCACACAGGAAACAGACCATGAGCTATCCGGAAAAATTTGAAGGC |
| *ycjV-ADH6*-a | AAACAACAGATAAAACGAAAGGCCCAGTCTTTCGACTGAGCCTTTCGTTTTATTTGTTAATCGCTAAATTCTTTATCATAGCCCACCAG |
| pGRB-*molR*-s | AGTCCTAGGTATAATACTAGTTGGCATTGCACCTAGCTACTGTTTTAGAGCTAGAA |
| pGRB-*molR*-a | TTCTAGCTCTAAAACAGTAGCTAGGTGCAATGCCAACTAGTATTATACCTAGGACT |
| *molR*-up-s | GTTGTTTTCTTGCGATTTTGTCTCTCTC |
| *molR*-up-a | AATTGTTATCCGCTCACAATTCCACACATTATACGAGCCGGATGATTAATTGTCAAGAAAGCGGCGATGACTTAACG |
| *molR*-dw-s | CCTTTCGTTTTATCTGTTGTTTGTCGGTGAACGCTCTCCTGAGTAGGACAAATCCCGAAAAACCAGAAATTGCAC |
| *molR*-dw-a | TTTTGCCCAAGAAGTTCCGTCA |
| *molR-EcHpaBC*-s | AATGTGTGGAATTGTGAGCGGATAACAATTTCACACAGGAAACAGACCATGAAACCAGAAGATTTCCGCGC |
| *molR-EcHpaBC*-a | GATAAAACGAAAGGCCCAGTCTTTCGACTGAGCCTTTCGTTTTATTTGTTAAATCGCAGCTTCCATTTCCAGC |
| pGRB-*yghX*-s | AGTCCTAGGTATAATACTAGTTATATCGCCCTGGCACCTGAGTTTTAGAGCTAGAA |
| pGRB-*yghX*-a | TTCTAGCTCTAAAACTCAGGTGCCAGGGCGATATAACTAGTATTATACCTAGGACT |
| *yghX*-up-s | TCAAACGCTTTACGCAGGAT |
| *yghX*-up-a | AATTGTTATCCGCTCACAATTCCACACATTATACGAGCCGGATGATTAATTGTCAAGCTCATCTTTGCGGGCTT |
| *yghX*-dw-s | AAAGACTGGGCCTTTCGTTTTATCTGTTGTTTGTCGGTGAACGCTCTCCTGAGTAGGACAAATATCCGCAAGCGACAGGC |
| *yghX*-dw-a | CGTTGATTCGGGTGTCCAG |
| *yghX-ARO10-ADH6*-s | TGTGTGGAATTGTGAGCGGATAACAATTTCACACAGGAAACAGACCATGGCGCCGGTGACCATT |
| *yghX-ARO10-ADH6*-a | AAACAACAGATAAAACGAAAGGCCCAGTCTTTCGACTGAGCCTTTCGTTTTATTTGTTAATCGCTAAATTCTTTATCATAGCCCACCAG |
| pGRB-*ydeU*-s | AGTCCTAGGTATAATACTAGTATTGCAGCTAGGCGGCGATTGTTTTAGAGCTAGAA |
| pGRB-*ydeU*-a | TTCTAGCTCTAAAACAATCGCCGCCTAGCTGCAATACTAGTATTATACCTAGGACT |
| *ydeU*-up-s | CAACGATTCCGCGGCGTAT |
| *ydeU*-up-a | AAATTGTTATCCGCTCACAATTCCACACATTATACGAGCCGGATGATTAATTGTCAAACCGTTGACCGTTTGTGCA |
| *ydeU*-dw-s | CCTTTCGTTTTATCTGTTGTTTGTCGGTGAACGCTCTCCTGAGTAGGACAAATATACTCAGGGCATGCTGGGA |
| *ydeU*-dw-a | GCGTCGCATAGCGTCATTGA |
| *ydeU-ARO10-ADH6-EcHpaBC*-s | TGTGTGGAATTGTGAGCGGATAACAATTTCACACAGGAAACAGACCATGGCGCCGGTGACCATT |
| *ydeU-ARO10-ADH6-EcHpaBC*-a | GATAAAACGAAAGGCCCAGTCTTTCGACTGAGCCTTTCGTTTTATTTGTTAAATCGCAGCTTCCATTTCCAGC |
| pGRB-*mbhA*-s | AGTCCTAGGTATAATACTAGTTACCGGGCATACCGATGCGAGTTTTAGAGCTAGAA |
| pGRB-*mbhA*-a | TTCTAGCTCTAAAACTCGCATCGGTATGCCCGGTAACTAGTATTATACCTAGGACT |
| *mbhA*-up-s | GCCAGCACGAACATAATCCC |
| *mbhA*-up-a | AATTGTTATCCGCTCACAATTCCACACATTATACGAGCCGGATGATTAATTGTCAACACGGTGGCAGGTTTTGG |
| *mbhA*-dw-s | AAAGACTGGGCCTTTCGTTTTATCTGTTGTTTGTCGGTGAACGCTCTCCTGAGTAGGACAAATGACCAAAAGTGCGTCCGATAC |
| *mbhA*-dw-a | CGGCGTAATCACAAACTGGC |
| pGRB-*rph*-s | AGTCCTAGGTATAATACTAGTTGCGACGTGCTTCAGGCTGAGTTTTAGAGCTAGAA |
| pGRB-*rph*-a | TTCTAGCTCTAAAACTCAGCCTGAAGCACGTCGCAACTAGTATTATACCTAGGACT |
| *rph*-up-s | ATAGCGCAGGGTACATTCCACTTTAC |
| *rph*-up-a | AATTGTTATCCGCTCACAATTCCACACATTATACGAGCCGGATGATTAATTGTCAACCTTCTTCAATAGAGGCGGTACACAAC |
| *rph*-dw-s | TGGGCCTTTCGTTTTATCTGTTGTTTGTCGGTGAACGCTCTCCTGAGTAGGACAAATTGCCGCAGAGACCGACATGAA |
| *rph*-dw-a | ACAGCGGTTGTGGTGGCAATC |
| pGRB-*yciQ*-s | AGTCCTAGGTATAATACTAGTAAACAACGTTTCTTGCCTCAGTTTTAGAGCTAGAA |
| pGRB-*yciQ*-a | TTCTAGCTCTAAAACTGAGGCAAGAAACGTTGTTTACTAGTATTATACCTAGGACT |
| *yciQ*-up-s | TTACTTGAAGCATTGGGCGAAC |
| *yciQ*-up-a | AATTGTTATCCGCTCACAATTCCACACATTATACGAGCCGGATGATTAATTGTCAACCAGTCAAGATGCCAGGGTTC |
| *yciQ*-dw-s | AAAGACTGGGCCTTTCGTTTTATCTGTTGTTTGTCGGTGAACGCTCTCCTGAGTAGGACAAATGTCTGACAAGAACCAGCAAATCCT |
| *yciQ*-dw-a | ATAGCTTCACCGTGGGCATAAC |
| pGRB-*gapC*-s | AGTCCTAGGTATAATACTAGTTACCTCCGCCGAGAAATCGCGTTTTAGAGCTAGAA |
| pGRB-*gapC*-a | TTCTAGCTCTAAAACGCGATTTCTCGGCGGAGGTAACTAGTATTATACCTAGGACT |
| *gapC*-up-s | TGGGAAGAAACCACGAAACTC |
| *gapC*-up-a | AATTGTTATCCGCTCACAATTCCACACATTATACGAGCCGGATGATTAATTGTCAATGTTTCAGCAGGTAGGCGAGA |
| *gapC*-dw-s | AAAGACTGGGCCTTTCGTTTTATCTGTTGTTTGTCGGTGAACGCTCTCCTGAGTAGGACAAATAAAACGGTCGCCTGGTACG |
| *gapC*-dw-a | TTATCCGCCGACATTGCTG |
| pGRB-*yfaS*-s | AGTCCTAGGTATAATACTAGTACCGTTCGAGCCGCAGCAAGGTTTTAGAGCTAGAA |
| pGRB-*yfaS*-a | TTCTAGCTCTAAAACCTTGCTGCGGCTCGAACGGTACTAGTATTATACCTAGGACT |
| *yfaS*-up-s | GAAGCGCCACGAAAACTGACA |
| *yfaS*-up-a | GTTATCCGCTCACAATTCCACACATTATACGAGCCGGATGATTAATTGTCAACGAGATTTGCCGTACCAGTTATCC |
| *yfaS*-dw-s | TTTCGTTTTATCTGTTGTTTGTCGGTGAACGCTCTCCTGAGTAGGACAAATACAACATATATCGCCAGAACGTTCA |
| *yfaS*-dw-a | TTAACTGCACGCGGGCATTTTTTA |
| pGRB-*pntAB*-s | AGTCCTAGGTATAATACTAGTTTAAGTAGTGATTCGTGCCGGTTTTAGAGCTAGAA |
| pGRB-*pntAB*-a | TTCTAGCTCTAAAACCGGCACGAATCACTACTTAAACTAGTATTATACCTAGGACT |
| *pntAB*-up-s | CCAGGTACTGGTATTGTTATTAACG |
| *pntAB*-up-a | GTTATCCGCTCACAATTCCACACATTATACGAGCCGGATGATTAATTGTCAAGCTGATACGGCTGTTTTTTAAGC |
| *pntAB*-dw-s | CGTATAATGTGTGGAATTGTGAGCGGATAACAATTTCACACAGGAAACAGACCATGCGAATTGGCATACCAAGAG |
| *pntAB*-dw-a | CCTCTTTAAAATCCAGCTCGAGG |
| pGRB-*ycgH*-s | AGTCCTAGGTATAATACTAGTATGCGTCTGAACGACCGTGCGTTTTAGAGCTAGAA |
| pGRB-*ycgH*-a | TTCTAGCTCTAAAACGCACGGTCGTTCAGACGCATACTAGTATTATACCTAGGACT |
| *ycgH*-up-s | TAAACTCGTCAGCGGCACAA |
| *ycgH*-up-a | AATTGTTATCCGCTCACAATTCCACACATTATACGAGCCGGATGATTAATTGTCAAGGTAGGCGTTTCTGTTGATTCTG |
| *ycgH*-dw-s | AAAGACTGGGCCTTTCGTTTTATCTGTTGTTTGTCGGTGAACGCTCTCCTGAGTAGGACAAATGCGTGTCGGATTATCGTTCG |
| *ycgH*-dw-a | GATTCAGGTTGCCATTTACGC |
| pGRB-*insB*-s | AGTCCTAGGTATAATACTAGTGTGCTAAATCACGTCAGCGCGTTTTAGAGCTAGAA |
| pGRB-*insB*-a | TTCTAGCTCTAAAACGCGCTGACGTGATTTAGCACACTAGTATTATACCTAGGACT |
| *insB*-up-s | GGATGGAGCCTCTGCTTCTGG |
| *insB*-up-a | TGTGTGAAATTGTTATCCGCTCACAATTCCACACATTATACGAGCCGGATGATTAATTGTCAACGGTTGTATGCGCGAGGTTAC |
| *insB*-dw-s | AAAGACTGGGCCTTTCGTTTTATCTGTTGTTTGTCGGTGAACGCTCTCCTGAGTAGGACAAATGGTGGAGTTGCATGACAAGGTC |
| *insB*-dw-a | GGTGGGCGTGCTGAATAAGAAG |
| pGRB-*ilvG*-s | AGTCCTAGGTATAATACTAGTTATCGGCACTGACGCATTTCGTTTTAGAGCTAGAA |
| pGRB-*ilvG*-a | TTCTAGCTCTAAAACGAAATGCGTCAGTGCCGATAACTAGTATTATACCTAGGACT |
| *ilvG*-up-s | ACCGAGGAGCAGACAATGAATAA |
| *ilvG*-up-a | AATTGTTATCCGCTCACAATTCCACACATTATACGAGCCGGATGATTAATTGTCAAGGTGATGGCAACAACAGGGATGGA |
| *ilvG*-dw-s | AAAGACTGGGCCTTTCGTTTTATCTGTTGTTTGTCGGTGAACGCTCTCCTGAGTAGGACAAATCTATCTACGCGCCGTTGTTGT |
| *ilvG*-dw-a | GCGCTGGCTAACATGAGGAA |
| ilv*G-ribA*-s | TGGAATTGTGAGCGGATAACAATTTCACACAGGAAACAGACCATGTTTCATCCGATAGAAGAAGCACTG |
| *ilvG-ribC*-a | CAGTCTTTCGACTGAGCCTTTCGTTTTATTTGTTATTTCCGCAAATTGCTGAAATAACGG |
| pGRB-*ygaY*-s | AGTCCTAGGTATAATACTAGTCACTGATGGCGCTGGCATTAGTTTTAGAGCTAGAA |
| pGRB-*ygaY*-a | TTCTAGCTCTAAAACTAATGCCAGCGCCATCAGTGACTAGTATTATACCTAGGACT |
| *ygaY*-up-s | CCTACAAACCACATCGCACATT |
| *ygaY*-up-105-a | CAGTGCTTCTTCTATCGGATGAAACATGGTCTGTTTCCTGCTAGCATAGTACCTAGGACTGAGCTAGCCGTAAAACACCGAAGCAAC |
| *ygaY*-dw-s | AGGCTCAGTCGAAAGACTGGGCCTTTCGTTTTATCTGTTGTTTGTCGGTGAACGCTCTCCTGAGTAGGACAAATCTATCTACGCGCCGTTGTTGT |
| *ygaY*-dw-a | GGAGTAGGGCTTTCCATAGAGTGT |
| pGRB-*ylbE*-s | AGTCCTAGGTATAATACTAGTGCGCCTGTAAACACTCCGCA GTTTTAGAGCTAGAA |
| pGRB-*ylbE*-a | TTCTAGCTCTAAAACTGCGGAGTGTTTACAGGCGCACTAGTATTATACCTAGGACT |
| *ylbE*-up-s | ATTGCGGTCGTGAGTTCGCTA |
| *ylbE*-up-101-a | TCCAGTGCTTCTTCTATCGGATGAAACATGGTCTGTTTCCTGCTAGCATAATACCTAGGACTGAGCTAGCTGTAAATTGTTCGATAACCGCAGCATTGG |
| *ylbE*-dw-s | TTCAGCAATTTGCGGAAATAACAAATAAAACGAAAGGCTCAGTCGAAAGACTGGGCCTTTCGTTTTATCTGTTGTTTGTCGGTGAACGCTCTCCTGAGTAGGACAAATCGCTGGCGTGCTTTGAACA |
| *ylbE*-dw-a | TGGTCGTTCATTGCCGCATAA |
| pGRB-*yjgX*-s | AGTCCTAGGTATAATACTAGTTGGCCATTGCGGATGGTTAAGTTTTAGAGCTAGAA |
| pGRB-*yjgX*-a | TTCTAGCTCTAAAACTTAACCATCCGCAATGGCCAACTAGTATTATACCTAGGACT |
| *yjgX*-up-s | GGAAGTCAACGGGTTATGCGG |
| *yjgX*-up-100-a | AGTGCTTCTTCTATCGGATGAAACATGGTCTGTTTCCTGCTAGCACTGTACCTAGGACTGAGCTAGCCGTCAAAAAATCACCACGAATACCAGAATCG |
| *yjgX*-dw-s | CTCAGTCGAAAGACTGGGCCTTTCGTTTTATCTGTTGTTTGTCGGTGAACGCTCTCCTGAGTAGGACAAATACAGTGTCTTCCCTGAGCC |
| *yjgX*-dw-a | GGCGAAGGATACCATCAAGCTG |

2. Synthetic gene sequences

2.1 KpHpaBC

ATGAAACCGGAAGATTTTCGCGCGGATGCGAAACGCCCGCTGACCGGCGAAGAATATCTGAAAAGCCTGCAAGATGGCCGCGAAATTTATATTTATGGCGAACGCGTGAAAGATGTTACCACCCATCCGGCGTTTCGCAACGCGGCCGCGAGCGTGGCGCAGCTGTATGATGCGCTGCATAAACCGGAAATGCAAGATAGCCTGTGCTGGGGCACCGATACCGGCAGCGGCGGCTATACCCATAAATTTTTTCGCGTGGCGAAAAGCGCGGATGATCTGCGTCAGCAGCGCGATGCGATTGCGGAATGGAGCCGCCTGAGCTATGGCTGGATGGGCCGCACCCCGGATTATAAAGCGGCGTTTGGCTGCGCGCTGGGCGCGAACCCGGCGTTTTATGGTCAGTTTGAACAGAACGCGCGCAACTGGTATACCCGCATTCAAGAAACCGGCCTGTATTTTAACCATGCGATTGTGAACCCGCCGATTGATCGCCATAAACCGGCGGATGAAGTGAAAGATGTGTATATTAAACTGGAAAAAGAAACCGATGCGGGCATTATTGTGAGCGGCGCGAAAGTGGTGGCGACCAACAGCGCGCTGACCCATTATAACATGATTGGCTTTGGCAGCGCGCAAGTGATGGGCGAAAACCCGGATTTTGCGCTGATGTTTGTGGCGCCGATGGATGCGGAAGGCGTGAAACTGATTAGCCGCGCGAGCTATGAAATGGTGGCGGGCGCGACCGGCAGCCCGTATGATTATCCGCTGAGCAGCCGCTTTGATGAAAACGATGCGATTCTGGTGATGGATAAAGTGCTGATTCCGTGGGAAAACGTGCTGATTTATCGCGATTTTGATCGCTGCCGCCGCTGGACCATGGAAGGCGGCTTTGCGCGCATGTATCCGCTGCAAGCGTGCGTGCGCCTGGCGGTGAAACTGGATTTTATTACCGCGCTGCTGAAACGCAGCCTGGAATGCACCGGCACCCTGGAATTTCGCGGCGTGCAAGCGGATCTGGGCGAAGTGGTGGCGTGGCGCAACATGTTTTGGGCGCTGAGCGATAGCATGTGCAGCGAAGCGACCCCGTGGGTGAACGGCGCGTGGCTGCCGGATCATGCGGCGCTGCAGACCTATCGCGTGATGGCGCCAATGGCGTACGCGAAAATTAAAAACATTATTGAACGCAACGTGACGAGCGGCCTGATTTATCTGCCGAGCAGCGCGCGCGATCTGAACAACCCGCAGATTGATCAGTATCTGGCGAAATATGTGCGCGGCAGCAACGGCATGGATCATGTGGAACGCATTAAAATTCTGAAACTGATGTGGGATGCGATTGGCAGCGAATTTGGCGGCCGCCATGAACTGTATGAAATTAACTATAGCGGCAGCCAAGATGAAATTCGCCTGCAGTGCCTGCGCCAAGCGCAGAGCAGCGGCAACATGGATAAAATGATGGCGATGGTGGATCGCTGCCTGAGCGAATATGATCAGAACGGCTGGACCGTGCCGCATCTGCATAACAACGCGGATATTAACATGCTGGATAAACTGCTGAAATAATTCACAGCGGGAGGTCATAATGCAGCTGGATGAACAGCGCCTGCGCTTTCGCGATGCCATGGCGAGTCTGAGCGCGGCCGTGAACGTTGTTACGACGGCGGGCGAAGCCGGCCGCTGCGGCATTACCGCGACCGCGGTGTGCAGCGTGACCGATACCCCGCCGAGCGTGATGGTGTGCATCAACGCGAACAGCGCGATGAACCCGGTGTTTCAAGGCAACGGCAAACTGTGCATTAATGTGCTGAACCATGAACAAGAAATTATGGCGCGCCATTTTGCGGGCATGACCGGCGTGACCATGGAAGAACGCTTTGCCCTGAGCGGTTGGCAGCAAGGCGCGCTGGGCCAACCGGTGCTGAAAGGCAGCCTGGCGAGCCTGGAAGGCGAAATTAGCCAAGTGCAGACCATTGGCACCCATCTGGTGTATCTGGTGGAAATTCGCAACATTACCCTGAGCCCGCAAGGCCATGGCCTGATTTATTTTAAACGCCGCTTTCATCCGGTGATGATGGAAATGGAAGTGGCGGTGTAA

2.2 PaHpaBC

ATGAAGCCAGAAGACTTCAGAGCTTCTGCTACTAGACCATTCACTGGTGAAGAATACTTGGCTTCTTTGAGAGACGACAGAGAAATCTACATCTACGGTGACAGAGTTAAGGACGTTACTTCTCACCCAGCTTTCAGAAACGCTGCTGCTTCTATGGCTAGATTGTACGACGCTTTGCACGACCCACAATCTAAGGAAAAGTTGTGTTGGGAAACTGACACTGGTAACGGTGGTTACACTCACAAGTTCTTCAGATACGCTAGATCTGCTGACGAATTGAGACAACAAAGAGACGCTATCGCTGAATGGTCTAGATTGACTTACGGTTGGATGGGTAGAACTCCAGACTACAAGGCTGCTTTCGGTTCTGCTTTGGGTGCTAACCCAGGTTTCTACGGTAGATTCGAAGACAACGCTAAGACTTGGTACAAGAGAATCCAAGAAGCTTGTTTGTACTTGAACCACGCTATCGTTAACCCACCAATCGACAGAGACAAGCCAGTTGACCAAGTTAAGGACGTTTTCATCTCTGTTGACGAAGAAGTTGACGGTGGTATCGTTGTTTCTGGTGCTAAGGTTGTTGCTACTAACTCTGCTTTGACTCACTACAACTTCGTTGGTCAAGGTTCTGCTCAATTGTTGGGTGACAACACTGACTTCGCTTTGATGTTCATCGCTCCAATGAACACTCCAGGTATGAAGTTGATCTGTAGACCATCTTACGAATTGGTTGCTGGTATCGCTGGTTCTCCATTCGACTACCCATTGTCTTCTAGATTCGACGAAAACGACGCTATCTTGGTTATGGACAAGGTTTTCATCCCATGGGAAAACGTTTTGATCTACAGAGACTTCGAAAGATGTAAGCAATGGTTCCCACAAGGTGGTTTCGGTAGATTGTTCCCAATGCAAGGTTGTACTAGATTGGCTGTTAAGTTGGACTTCATCACTGGTGCTTTGTACAAGGCTTTGCAATGTACTGGTTCTTTGGAATTCAGAGGTGTTCAAGCTCAAGTTGGTGAAGTTGTTGCTTGGAGAAACTTGTTCTGGTCTTTGACTGACGCTATGTACGGTAACGCTTCTGAATGGCACGGTGGTGCTTTCTTGCCATCTGCTGAAGCTTTGCAAGCTTACAGAGTTTTGGCTCCACAAGCTTACCCAGAAATCAAGAAGACTATCGAACAAGTTGTTGCTTCTGGTTTGATCTACTTGCCATCTGGTGTTAGAGACTTGCACAACCCACAATTGGACAAGTACTTGTCTACTTACTGTAGAGGTTCTGGTGGTATGGGTCACAGAGAAAGAATCAAGATCTTGAAGTTGTTGTGGGACGCTATCGGTTCTGAATTCGGTGGTAGACACGAATTGTACGAAATCAACTACGCTGGTTCTCAAGACGAAATCAGAATGCAAGCTTTGAGACAAGCTATCGGTTCTGGTGCTATGAAGGGTATGTTGGGTATGGTTGAACAATGTATGGGTGACTACGACGAAAACGGTTGGACTGTTCCACACTTGCACAACCCAGACGACATCAACGTTTTGGACAGAATCAGACAATAAATGTCTCAATTGGAACCAAGACAACAAGCTTTCAGAAACGCTATGGCTCACTTGTCTGCTGCTGTTAACGTTATCACTTCTAACGGTCCAGCTGGTAGATGTGGTATCACTGCTACTGCTGTTTGTTCTGTTACTGACTCTCCACCAACTTTGATGTTGTGTATCAACAGAAACTCTGAAATGAACACTGTTTTCAAGGCTAACGGTAGATTGTGTGTTAACGTTTTGTCTGGTGAACACGAAGAAGTTGCTAGACACTTCGCTGGTATGACTGAAGTTCCAATGGAAAGAAGATTCGCTTTGCACGACTGGAGAGAAGGTTTGGCTGGTTTGCCAGTTTTGCACGGTGCTTTGGCTAACTTGCAAGGTAGAATCGCTGAAGTTCAAGAAATCGGTACTCACTCTGTTTTGTTGTTGGAATTGGAAGACATCCAAGTTTTGGAACAAGGTGACGGTTTGGTTTACTTCTCTAGATCTTTCCACAGATTGCAATGTCCAAGAAGAGCTGCTTAA

2.3 ARO10

ATGGCGCCGGTGACCATTGAAAAATTTGTGAACCAAGAAGAACGCCATCTGGTGAGCAACCGCAGCGCGACCATTCCGTTTGGCGAATATATTTTTAAACGCCTGCTGAGCATTGATACCAAAAGCGTGTTTGGCGTGCCGGGCGATTTTAACCTGAGCCTGCTGGAATATCTGTATAGCCCGAGCGTGGAAAGCGCGGGCCTGCGCTGGGTGGGCACCTGCAACGAACTGAACGCGGCGTATGCGGCGGATGGCTATAGCCGCTATAGCAACAAAATTGGCTGCCTGATTACCACCTATGGCGTGGGCGAACTGAGCGCGCTGAACGGCATTGCGGGCAGCTTTGCGGAAAACGTGAAAGTGCTGCATATTGTGGGCGTGGCGAAAAGCATTGATAGCCGCAGCAGCAACTTTAGCGATCGCAACCTGCATCATCTGGTGCCGCAGCTGCATGATAGCAACTTTAAAGGCCCGAACCATAAAGTGTATCATGATATGGTGAAAGATCGCGTGGCGTGCAGCGTGGCGTATCTGGAAGATATTGAAACCGCGTGCGATCAAGTGGATAACGTGATTCGCGATATTTATAAATATAGCAAACCGGGCTATATTTTTGTGCCGGCGGATTTTGCGGATATGAGCGTGACCTGCGATAACCTGGTGAACGTGCCGCGCATTAGTCAGCAAGATTGCATTGTGTATCCGAGCGAAAATCAGCTGAGCGATATTATTAACAAAATTACGAGCTGGATTTATAGCAGCAAAACCCCGGCGATTCTGGGCGATGTGCTGACCGATCGCTATGGCGTGAGCAACTTTCTGAACAAACTGATTTGCAAAACCGGCATTTGGAACTTTAGCACCGTGATGGGCAAAAGCGTGATTGATGAAAGCAACCCGACCTATATGGGTCAGTATAACGGCAAAGAAGGCCTGAAACAAGTGTATGAACATTTTGAACTGTGCGATCTGGTGCTGCATTTTGGCGTGGATATTAACGAAATTAACAACGGCCATTATACCTTTACCTATAAACCGAACGCGAAAATTATTCAGTTTCATCCGAACTATATTCGCCTGGTGGATACCCGCCAAGGCAACGAACAGATGTTTAAAGGCATTAACTTTGCGCCGATTCTGAAAGAACTGTATAAACGCATTGATGTGAGCAAACTGAGCCTGCAGTATGATAGCAACGTGACGCAGTATACCAACGAAACCATGCGCCTGGAAGATCCGACCAACGGTCAGAGCAGCATTATTACCCAAGTGCATCTGCAGAAAACCATGCCGAAATTTCTGAACCCGGGCGATGTGGTTGTGTGCGAAACCGGCAGCTTTCAGTTTAGCGTGCGCGATTTTGCGTTTCCGAGTCAGCTGAAATATATTAGCCAAGGCTTTTTTCTGAGCATTGGCATGGCGCTGCCGGCGGCGCTGGGCGTGGGCATTGCGATGCAAGATCATAGCAACGCGCATATTAACGGCGGCAACGTGAAAGAAGATTATAAACCGCGCCTGATTCTGTTTGAAGGCGATGGCGCGGCGCAGATGACCATTCAAGAACTGAGCACCATTCTGAAATGCAACATTCCGCTGGAAGTGATTATTTGGAACAATAACGGCTATACCATTGAACGCGCGATTATGGGCCCGACCCGCAGCTATAACGATGTGATGAGCTGGAAATGGACCAAACTGTTTGAAGCGTTTGGCGATTTTGATGGCAAATATACCAACAGCACCCTGATTCAGTGCCCGAGCAAACTGGCGCTGAAACTGGAAGAACTGAAAAACAGCAACAAACGCAGCGGCATTGAACTGCTGGAAGTGAAACTGGGCGAACTGGATTTTCCGGAACAGCTGAAATGCATGGTGGAAGCGGCCGCGCTGAAACGCAACAAAAAATAA

2.4 ADH6

ATGAGCTATCCGGAAAAATTTGAAGGCATTGCGATTCAGAGCCATGAAGATTGGAAAAACCCGAAAAAAACCAAATATGATCCGAAACCGTTTTATGATCATGATATTGATATCAAAATTGAGGCGTGCGGCGTGTGCGGCAGCGATATTCATTGCGCGGCGGGCCATTGGGGCAACATGAAAATGCCGCTGGTGGTGGGCCATGAAATTGTGGGCAAAGTGGTGAAACTGGGCCCGAAAAGCAACAGCGGCCTGAAAGTGGGTCAGCGCGTGGGCGTGGGCGCGCAAGTGTTTAGCTGCCTGGAATGCGATCGCTGCAAAAACGATAACGAACCGTATTGCACCAAATTTGTGACCACCTATAGTCAGCCGTATGAAGATGGCTATGTGAGCCAAGGCGGCTATGCGAACTATGTGCGCGTGCATGAACATTTTGTGGTGCCGATTCCGGAAAACATTCCGAGCCATCTGGCGGCGCCGCTGCTGTGCGGCGGCCTGACCGTGTATAGCCCGCTGGTGCGCAACGGCTGCGGCCCGGGCAAAAAAGTGGGCATTGTGGGCCTGGGCGGCATTGGCAGCATGGGCACCCTGATTAGCAAAGCGATGGGCGCGGAAACCTATGTGATTAGCCGCAGCAGCCGCAAACGCGAAGATGCGATGAAAATGGGCGCGGATCATTATATTGCGACCCTGGAAGAAGGCGATTGGGGCGAAAAATATTTTGATACCTTTGATCTGATTGTGGTGTGCGCGAGCAGCCTGACCGATATTGATTTTAACATTATGCCGAAAGCGATGAAAGTGGGCGGCCGCATTGTGAGCATTAGCATTCCGGAACAGCATGAAATGCTGAGCCTGAAACCGTATGGCCTGAAAGCGGTTAGCATTAGTTATAGCGCGCTGGGCAGCATTAAAGAACTGAATCAGCTGCTGAAACTGGTGAGCGAAAAAGATATTAAAATTTGGGTGGAAACCCTGCCGGTGGGCGAAGCGGGCGTGCATGAAGCGTTTGAACGCATGGAAAAAGGCGATGTGCGCTATCGCTTTACCCTGGTGGGCTATGATAAAGAATTTAGCGATTAA
